# Support vector regression-guided unravelling: antioxidant capacity and quantitative structure-activity relationship predict reduction and promotion effects of flavonoids on acrylamide formation

**DOI:** 10.1038/srep32368

**Published:** 2016-09-02

**Authors:** Mengmeng Huang, Yan Wei, Jun Wang, Yu Zhang

**Affiliations:** 1 Zhejiang Key Laboratory for Agro-Food Processing, Zhejiang R & D Center for Food Technology and Equipment, Fuli Institute of Food Science, Zhejiang University, Hangzhou 310058, Zhejiang, China; 2 Department of Food Science and Nutrition, College of Biosystems Engineering and Food Science, Zhejiang University, Hangzhou 310058, Zhejiang, China

## Abstract

We used the support vector regression (SVR) approach to predict and unravel reduction/promotion effect of characteristic flavonoids on the acrylamide formation under a low-moisture Maillard reaction system. Results demonstrated the reduction/promotion effects by flavonoids at addition levels of 1–10000 μmol/L. The maximal inhibition rates (51.7%, 68.8% and 26.1%) and promote rates (57.7%, 178.8% and 27.5%) caused by flavones, flavonols and isoflavones were observed at addition levels of 100 μmol/L and 10000 μmol/L, respectively. The reduction/promotion effects were closely related to the change of trolox equivalent antioxidant capacity (ΔTEAC) and well predicted by triple ΔTEAC measurements via SVR models (*R*: 0.633–0.900). Flavonols exhibit stronger effects on the acrylamide formation than flavones and isoflavones as well as their O-glycosides derivatives, which may be attributed to the number and position of phenolic and 3-enolic hydroxyls. The reduction/promotion effects were well predicted by using optimized quantitative structure-activity relationship (QSAR) descriptors and SVR models (*R*: 0.926–0.994). Compared to artificial neural network and multi-linear regression models, SVR models exhibited better fitting performance for both TEAC-dependent and QSAR descriptor-dependent predicting work. These observations demonstrated that the SVR models are competent for predicting our understanding on the future use of natural antioxidants for decreasing the acrylamide formation.

Acrylamide, a neurotoxin and probable human carcinogen, has attracted worldwide attention since high concentrations of acrylamide were discovered in thermally processing foods^1^,^2^. It is well-known that acrylamide is generated from the Maillard reaction in many carbohydrate-rich foods, particularly potato chips, crispy bread, cookies, breakfast cereal and coffee^3^,^4^. Based on these food matrices, scientists have conducted a great number of researches regarding the analysis of acrylamide and its substrates, formation mechanism of acrylamide, and its mitigation strategies^5^,^6^,^7^. Asparagine and acrolein pathways, and related precursors and intermediates (e.g., 3-aminopropionamide, Schiff base and Amadori rearrangement product) have been proposed to contribute to the generation of acrylamide^8^,^9^,^10^. Based on the acknowledged elucidation of acrylamide formation, increasing number of exogenous additives or chemical agents have been employed to mitigate the acrylamide formation, including organic acids, amino acids and mono- and divalent cations^11^.

Natural antioxidant herbs, especially polyphenol-rich extracts, were fully considered as effective agents for the mitigation of acrylamide in foods. Early study demonstrated that addition of raw materials from selected dietary herbs and their crude aqueous extracts could considerably reduce the acrylamide formation in both cookie and potato starch-based matrices^12^. Oral *et al.*^13^ found that all selected phenolic compounds and plant herbs except oleuropein decrease the acrylamide levels within the range of 30.8–85.0% in glycine-glucose and asparagine-fructose model systems, and reduce the acrylamide formation by 10.3–19.2% in biscuit. Antioxidants of bamboo leaves (AOB), sodium erythorbate, tea polyphenols, vitamin E, and *tert*-butyl hydroquinone were also used to reduce the formation of acrylamide during cookie processing and promisingly, AOB and vitamin E could exert their inhibition effects without the negative observation of sensory properties^14^.

The production of acrylamide during frying, baking and roasting needs a low-moisture medium. Thus, it is important to mimic the Maillard reaction under low-moisture conditions for in-depth investigations. Delgado *et al.*^15^ demonstrated that the moisture content, water activity and physical state of water may affect the mitigation of acrylamide during processing of tortilla chips. More studies are expected to clarify the formation mechanism of acrylamide via the effect of moisture and pursue the reduction of acrylamide under low-moisture reaction conditions. However, the dual effect of exogenous antioxidants on the acrylamide formation has been observed. First, natural antioxidants at their different addition levels exhibit different reduction or promotion effects on the acrylamide formation. The addition of green tea extract (0.25 g/100 g) and green coffee extract (1 g/100 g) induce dramatic increase of acrylamide, while the use of both antioxidants at other addition levels reduces the formation of acrylamide^16^. Second, strong antioxidant properties do not indicate a promising reduction effect. Cinnamic acid and some phenolic compounds added at 5 mmol/L display different antioxidant capacity, but all show no reduction effect on the net amount of acrylamide^17^. Therefore, both positive and negative effects by antioxidants need further scientific elucidation.

Flavonoids are polyphenolic compounds and widely distributed as secondary metabolites in plant kingdom, which have been classified as flavones, flavanones, flavanols, flavonols, anthocyanidines, isoflavonoids and neoflavonoids. Their biological, pharmacological and medicinal properties have been extensively reported elsewhere^18^. Nearly all groups of flavonoids are capable of acting as powerful chain-breaking antioxidants^19^. The antioxidant activity of flavonoids depends on the stable role in different matrices, as well as the number and position of hydroxyl substitutions in their structures^20^. Our pioneer study revealed that AOB could not only significantly reduce the acrylamide formation in potato-based foods but also retain original flavor and odor attributes, while the flavonoid ingredients of AOB play an important role in the reduction of acrylamide^21^. In a mechanistic view, flavonoids as polyphenols are capable of inhibiting the acrylamide formation via trapping carbonyl compounds or preventing lipid oxidation. However, some polyphenols including silymarin and curcumin are capable of providing carbonyl groups and thus promoting the conversion of asparagine into acrylamide^22^. In a kinetic view, we have recently unraveled the effect of flavonoids on the kinetic profiles of acrylamide in the Maillard reaction and indicated that flavonoids significantly suppress the formation-predominant stage of acrylamide during the kinetic process but do not affect its elimination-predominant process^23^. In a structural view, the role of characteristic antioxidant groups such as phenolic hydroxyls in the acrylamide generation and elimination needs to be further addressed. The quantitative structure-activity relationship (QSAR) approach is an acknowledged computational tool for evaluating the biological functions in a structural view. Especially, this chemometric method has been conducted in high-throughput screening of active ingredients from natural herbs or medical drugs^24^. Unfortunately, few studies have reported the use of QSAR approach for predicting the reduction and/or promotion of acrylamide by natural antioxidants yet.

Given antioxidant- and structure-dependent relationships, the antioxidant attributes of Maillard reaction products (MRP) and functional groups of flavonoids may be used for predicting the generation and elimination of acrylamide. Support vector machines (SVM) are powerful machine learning models with associated learning algorithms that analyze data used for classification and regression analysis^25^. As a “black box” modelling approach, the key features of SVM include the use of kernels, the absence of local minima, the sparseness of the solution and the capacity control achieved by optimizing the margin^26^, which may provide a modelling tool for predicting the profile of acrylamide generation and reduction. Unfortunately, we have not found any related predictive study using the support vector regression (SVR) approach yet.

The present work systematically evaluated the effect of 13 representative flavonoids, including flavones, flavonols and isoflavones, on the acrylamide formation via investigating of antioxidant- and structure-dependent relationships using the SVR approach under the low-moisture heating conditions, and predicted the contribution of antioxidant properties of MRP and chemical structures of flavonoid antioxidants to the inhibition or promotion on the acrylamide formation.

## Results and Discussion

### Dose-response effects of flavonoids on acrylamide formation under low-moisture system

**Figure 1:**
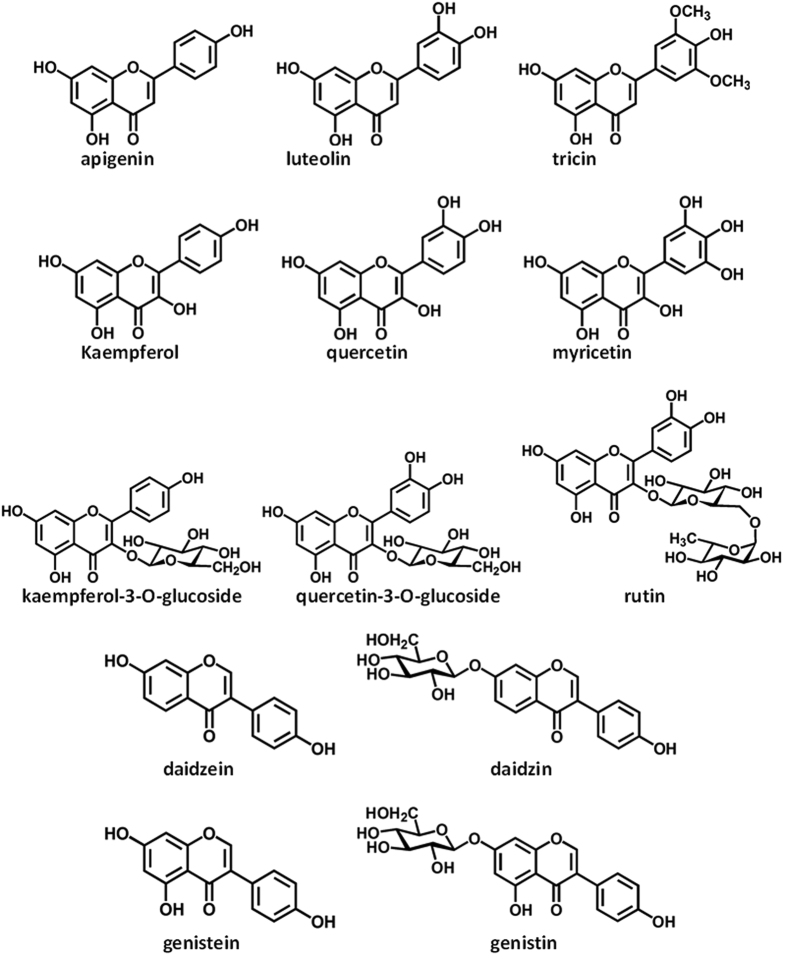
Chemical structures of selected flavones, flavonols and their 3-O-glycosides, and isoflavones.

**Figure 2:**
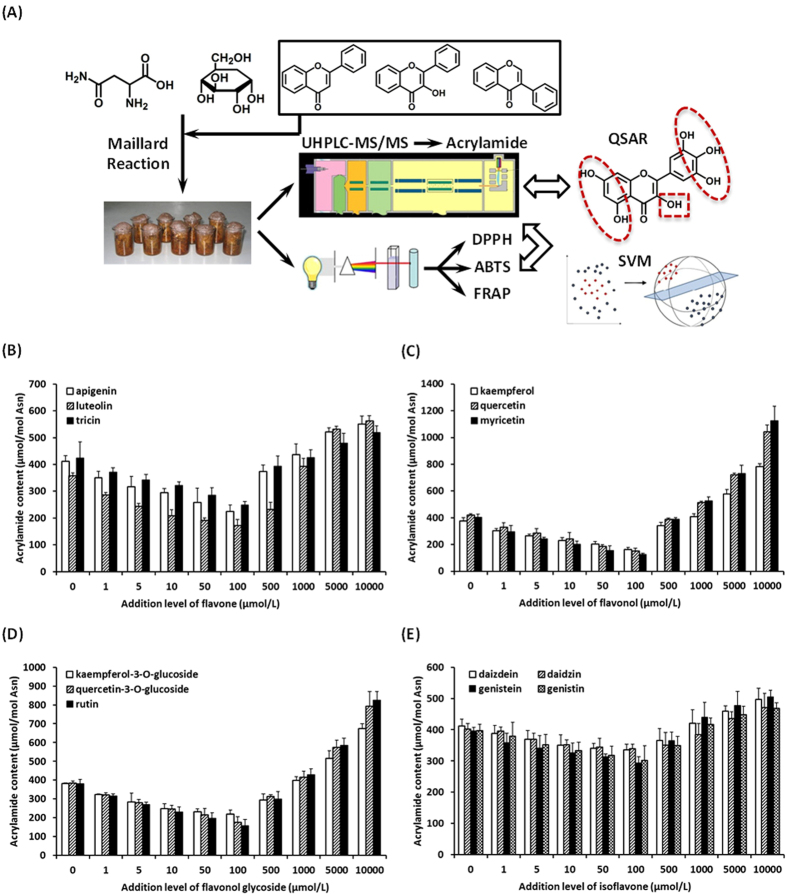
The inhibition and promotion effects of flavonoids on the acrylamide formation in a low-moisture Maillard reaction system. (**A**) The principle and procedure of current experimental design. (**B–E**) The dose-response bell-shaped correlation between the acrylamide level and the addition level of (**B**) flavones, (**C**) flavonols, (**D**) flavonol-3-O-glycosides, and (**E**) isoflavones. Data were shown in the form of mean ± SD (*n* = 3). ABTS, 2,2-azino-bis-3-ethylbenzothiazoline-6-sulphonic acid; Asn, asparagine; DPPH, 2,2-diphenyl-1-picrylhydrazyl; FRAP, ferric reducing antioxidant power; QSAR, quantitative structure activity relationship; SVM, support vector machine; UHPLC-MS/MS, ultra-high performance liquid chromatography tandem mass spectrometry.

In the present work, we selected some representative flavonoids as natural antioxidant agents, including flavones (apigenin, luteolin and tricin), flavonols (kaempferol, kaempferol-3-O-glucoside, quercetin, quercetin-3-O-glucoside, rutin and myricetin) and isoflavones (daidzein, genistein, daidzin and genistin) (Fig. 1). The Maillard reaction between asparagine and glucose in an equimolar level was conducted to investigate the effect of selected flavonoids on acrylamide formation in a low-moisture potato matrix through oven-heating treatments (Fig. 2A). Results of the dose-response study demonstrated a non-linear association of the acrylamide formation with the addition levels of flavonoids. Particularly, the reduction effect by flavonoids enhanced with the increment of their addition levels ranging from 1 to 100 μmol/L but declined afterwards when the addition level surpassed 100 μmol/L. Besides, the promotion effect on the acrylamide generation unexpectedly increased when the treatment levels of flavonoids rose from 500 to 10000 μmol/L. In detail, the inhibition rates of acrylamide generation induced by flavones, flavonols and isoflavones ranged 7.1–51.7%, 3.7–68.8% and 1.6–26.1%, respectively, while the promotion rates of acrylamide formation spanned 0.7–57.7%, 4.5–178.8% and 2.3–27.5% within the whole range of addition levels, respectively (Fig. 2B–E). Finally, 100 μmol/L was considered as the optimal effective concentration because flavonoids in this addition level exerted their maximal reduction effect.

Results of the dual effect on the acrylamide generation indicated that both prooxidant and antioxidant properties of selected flavonoids may occur during the dose-response study, the functionality of which mainly depends on the addition levels of flavonoid antioxidants. Similarly, previous study reported that the 1% use of aqueous extract of dittany, a phenol-rich natural product from the Labiatae family, reduces the acrylamide generation in wheat buns while the 10% use of the extract slightly promotes the acrylamide generation compared with the production of acrylamide in wheat buns without any dittany treatment^27^. In some cases, different kinds of antioxidant agents exert distinct effect on the acrylamide generation. The addition of chlorogenic acid significantly increases acrylamide formation and inhibits its elimination in the asparagine/glucose model system due to the enhancement of deamination from 3-aminopropionamide (3-APA) and the protection of acrylamide against the attack from free radicals^28^. Nevertheless, a nonlinear dose-response relationship between the addition levels of flavonoids and their reduction effects was demonstrated under the microwave heating model system. However, no promotion effect was observed within the whole range of the addition levels of flavonoids^29^. The different consequences at series of concentrations indicated the presence of multiple reactions between polyphenols and substrates of Maillard reaction^22^, which may be related to the addition levels of the additives as well as different heating treatment methods and types of reaction systems or food matrices.

Acrylamide is produced under the low-moisture condition during heat processing such as frying, baking and roasting. Recent study demonstrated that low levels of acrylamide is generated during microwave heating at low temperatures for a long time in soybean products due to its partial degradation, while acrylamide contents greatly increase with decreasing moisture content during infrared heating^30^. Similarly, our previous work showed that the addition of AOB or extract of green tea could reduce the acrylamide formation during the formation-predominant kinetic stage and prolong the progress of acrylamide production in the low-moisture medium^31^. The same trend was also reported in real food matrices, where acrylamide formation occurs to a large extent only when the moisture content is below 5%^32^. Thus, the low-moisture status for mimicking the acrylamide generation and elimination should be taken into consideration.

The dose-dependent reduction/promotion effect of flavonoids on the acrylamide formation may be due to various pathways in Maillard reaction. For example, trapping of carbonyl compounds and preventing against lipid oxidation may inhibit the acrylamide formation, while providing carbonyl groups, accelerating the conversion of 3-APA into acrylamide and inhibiting acrylamide elimination may enhance the acrylamide content in final MRP^22^. Flavonoids are capable of both interacting with some key intermediates such as 3-APA to reduce the acrylamide formation and triggering the degradation of sugars to enhance the acrylamide formation. The net results regarding reduction or promotion effect may depend on the addition levels of antioxidants^33^. Besides, the carbonyl group in the chemical structures of antioxidants may also interact with asparagine to generate acrylamide^34^.

Overall, the present work revealed that the use of flavonoids is in favor of reducing the acrylamide formation only when a fitting but not the minimal or maximal level (100 μmol/L) is employed. The antioxidant and prooxidant properties of flavonoids contributing to the reduction and promotion of acrylamide are relatively defined because they are transformed reversely under certain reaction conditions, such as different additions levels, water activity and heat-processing methods.

### Comparison of dual effects of flavonoids on acrylamide formation on a structural basis

Flavonoids are a considerably large family including many polyphenolic compounds with different chemical structures, which have significant impacts on the acrylamide formation. In the present work, the selected flavonoids present different flavonoid skeletons, including various numbers and positions of phenolic and alcoholic hydroxyls and related glycoside groups, which may be related to their reduction and promotion effects.

Flavonols, which contain the 3-enolic hydroxyl, presented superior reduction/promotion effect on the acrylamide formation to the effect of flavones when sharing the same flavonoid aglycone (Fig. 2B–D). Isoflavones, 3-isomers of flavones, exhibited the weakest reduction/promotion effect among the three categories of flavonoids when the same addition level was added (Fig. 2E). The differences of chemical structures may principally explain their distinct reduction/promotion effect on the acrylamide formation. Furthermore, results indicated that the sum of phenolic and 3-enolic hydroxyls of flavonoids may substantially contribute to their both reduction and promotion effects on acrylamide formation. We investigated the association of reduction/promotion effect of flavonoids with their phenolic and 3-enolic hydroxyls, and concluded that the enhancement of both inhibition and promotion effects is associated with increasing numbers of phenolic and 3-enolic hydroxyls at all addition levels of flavonoids (Table 1). For example, given the same flavonoid skeleton and the same addition level, the reduction/promotion effect of quercetin (inhibition rate: 21.8–63.9%, promotion rate: 22.5–148.2%), quercetin-3-O-glucoside (16.2–54.5%, 8.2–107.3%), genistein (8.1–26.1%, 11.0–27.5%) and genistin (4.6–23.9%, 5.0–18.1%), which present more phenolic hydroxyls, are higher than those of kaempferol (9.5–56.6%, 8.3–108.4%), kaempferol-3-O-glucoside (15.2–42.4%, 4.5–76.7%), daidzein (5.8–18.5%, 2.3–20.7%) and daidzin (1.6–15.5%, 0.7–17.1%), respectively.

In addition, we also observed that flavonoids exhibit better reduction/promotion effect on the acrylamide generation than the effect of their O-glycosides in the present work. Results indicated that all flavonoid O-glycosides, in which O-glycosides substitute the 7-phenolic hydroxyl of A-ring, 4′-phenolic hydroxyl of B-ring or 3- enolic hydroxyls of C-ring in corresponding flavonoid skeletons, exhibit weakened reduction/promotion effect on the acrylamide formation. This finding may be due to different numbers of phenolic hydroxyls or 3-enolic hydroxyl in flavonoid skeletons compared to their glycosides when the insertion of O-glycosides reduces 1 phenolic or 3-enolic hydroxyl^35^.

It is acknowledged that herbal extracts and phenolic compounds may affect the acrylamide formation both positively and negatively^22^. Unfortunately, few studies have reported the use of natural product additives for controlling the acrylamide formation in view of structure-activity relationship yet. Recent studies showed that the extracts from green tea, cinnamon and oregano reduce the acrylamide level in fried potatoes by 62%, 39% and 17%, respectively, whereas two plant extracts from thyme and bougainvillea have no significant influence on acrylamide formation in the same condition, which may be ascribed to their diverse chemical structures^36^. Our previous work revealed that flavone C-glycosides are more active than flavone O-glycosides for their reduction effect on the acrylamide formation when they possess the same aglycone skeleton, which may be related to their different patterns on the glycoside replacement of flavone skeletons^37^. Taken together, we concluded that the number and position of phenolic and 3-enolic hydroxyls but not alcoholic hydroxyls of flavonoids greatly contribute to the acrylamide generation, which needs to be further validated via the chemometric approach.

### SVM-guided unravelling of reduction/promotion effect of flavonoids on acrylamide formation via antioxidant properties of MRP

**Figure 3:**
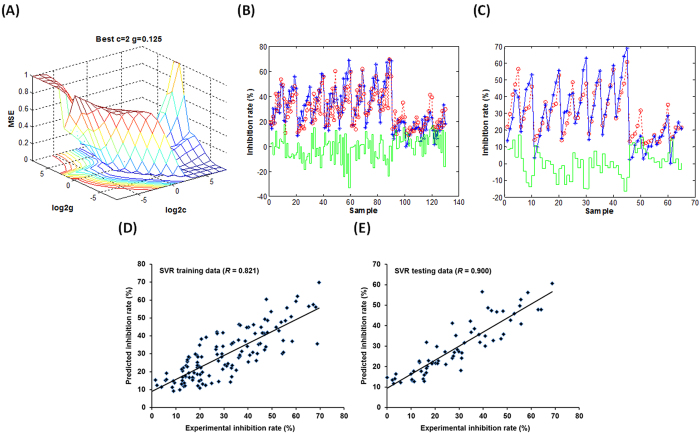
The establishment and performance of SVR models for predicting the reduction effect on the acrylamide formation via taking triple antioxidant measurements (DPPH, ABTS and FRAP assays) as variables when the addition levels of flavonoids ranged 1–100 μmol/L. (**A**) The optimization of SVR parameters *c* and *g*. (**B,C**), The fitting outcomes of (**B**) training data and (**C**) testing data via using the libsvm toolbox of the Matlab software. The red, blue and green lines indicate the predictive data, experimental data and predictive error, respectively. (**D,E**), The scatter plots exhibiting the association of predicted data with measured data for (**D**) training data and (**E**) testing data using the established SVR model.

**Figure 4:**
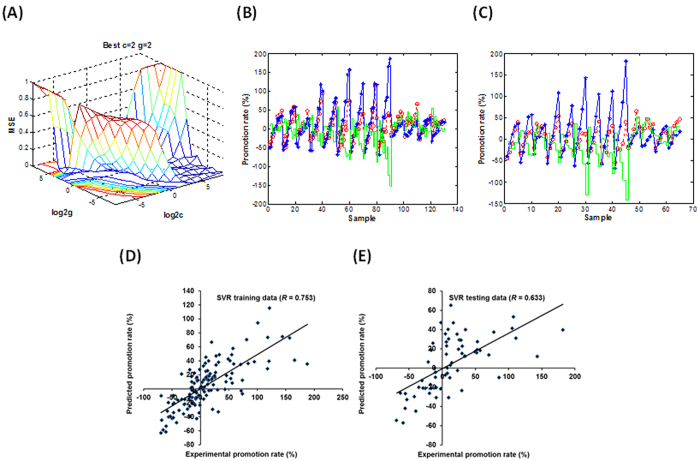
The establishment and performance of SVR models for predicting the promotion effect on the acrylamide formation via taking triple antioxidant measurements (DPPH, ABTS and FRAP assays) as variables when the addition levels of flavonoids ranged 100–10000 μmol/L. (**A**) The optimization of SVR parameters *c* and *g*. (**B,C**), The fitting outcomes of (**B**) training data and (**C**) testing data via using the libsvm toolbox of the Matlab software. The red, blue and green lines indicate the predictive data, experimental data and predictive error, respectively. (**D,E**), The scatter plots exhibiting the association of predicted data with measured data for (**D**) training data and (**E**) testing data using the established SVR model.

Considering the association of the change of acrylamide contents with the antioxidant characteristics of MRP, we investigated the antioxidant property of MRP via a triple evaluation system. The 2,2-diphenyl-1-picrylhydrazyl (DPPH), 2,2-azino-bis-3-ethylbenzothiazoline-6-sulphonic acid (ABTS) and ferric reducing antioxidant power (FRAP) assays were used to measure the trolox equivalent antioxidant capacity (TEAC) levels of MRP in all groups. DPPH, ABTS and FRAP assays are three representative evaluation methods that measure the antioxidant properties via the singe electron transfer induced elimination of radicals^38^,^39^, the mechanism of which could be used to elucidate the antioxidant role of some natural products such as flavonoids. A non-linear and bell-shaped dose-response relationship between the TEAC_DPPH_/TEAC_ABTS_/TEAC_FRAP_ values and the addition levels of flavonoids was observed (Supplementary Figs 1–4). To investigate and compare both non-linear effects and model fitting performance, SVR, artificial neural network (ANN) and multiple linear regression (MLR) methods were comparably employed to estimate both inhibition and promotion effects on the acrylamide formation using triple antioxidant measurements. A SVR principle was used to establish a regression model via considering the combination of ΔTEAC_DPPH_, ΔTEAC_ABTS_ and ΔTEAC_FRAP_ as independent variables and predict the inhibition/promotion rate of acrylamide formation. The standard epsilon (ξ) insensitive SVR model used in the present work sets an ξ tube around data points within which errors are discarded using an ξ insensitive loss function^40^. Under such situation, the selection criteria for an optimized SVR model mainly include the parameter optimization of cost of ξ-SVR (*c*) and γ-function of radial basis function (RBF) (*g*). The optimized process of SVR parameters (*c* and *g*) and the performance of SVR models established when the addition levels of flavonoids were 1–100 μmol/L were observed and shown in Fig. 3A–C. The scatter plots exhibiting the correlation between experimental and predictive results for both training and testing data set were shown in Fig. 3D–E. Similarly, the parameter optimization and the performance of SVR models and related correlation plots when the addition levels were 100–10000 μmol/L were shown in Fig. 4A–E. The performance of SVR models was checked via applying the 5-fold cross validation technique with its predictive ability and using the cross validation correlation coefficient (*Q*) and mean squared error (CV-MSE), which were shown in Table 2. The correlation coefficients (*R*) of established SVR models for predicting the inhibition/promotion rate affected by flavonoids (*C*_1_: 1–100 μmol/L and *C*_2_: 100–10000 μmol/L) were calculated as 0.900 and 0.633, respectively, indicating good fit for current TEAC-dependent prediction.

To compare with the SVR prediction, both ANN and MLR methods were successively applied to estimate inhibition/promotion rate for the acrylamide formation. As an alternative machine learning method, an ANN model is a multi-layer feed-forward network where the neurons are appropriately assigned into input, hidden and output layers. Such feed-forward neural network follows predesigned learning algorithm to process non-linear correlations^41^. In the present work, a back-propagation training algorithm was optimized according to our previous work^29^ and applied to the ANN regression. The fitting performance of ANN models when the addition levels of flavonoids fell into the scope of 1–100 μmol/L and 100–10000 μmol/L were shown in Supplementary Figs. 5 and 6, respectively. Compared to the predictive results using SVR models, the two correlations that currently established ANN models were used for predicting inhibition effect (*R* = 0.870) and promotion effect (*R* = 0.612) both appear weaker fitting outcomes (Table 2). Furthermore, current TEAC-dependent inhibition/promotion effect was also predicted by the routine MLR method. In detail, the MLR equations representing the association of both inhibition and promotion rates (*Y*_1_ and *Y*_2_) with antioxidant properties of MRP (*X*_1_, ΔTEAC_DPPH_; *X*_2_, ΔTEAC_ABTS_ and *X*_3_, ΔTEAC_FRAP_) were shown as follows:


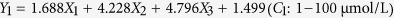



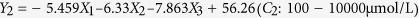


Considering the model performance, the evaluation of statistical variables including root mean square error (RMSE) and mean absolute percentage error (MAPE) also demonstrated that current optimized SVR models (*R*: 0.633–0.900) were appropriate for estimating the data of both inhibition and promotion effects from both training and testing sets, which were superior to that of both ANN model (*R*: 0.612–0.870) and MLR model (*R*: 0.631–0.898) (Table 2). The predictive performance of inhibition rates via all three predictive methods seems better than that of promotion rates. Besides the applications in the field of computational chemistry, SVM as a predictive guide tool has recently been employed in other researches such as antioxidant-related studies^42^,^43^. In this study, we used SVR models to connect inhibition and promotion effects on the acrylamide formation with antioxidant properties and offer a statistical support for the probable correlation. The current SVR models provide predictable approaches for estimating both inhibition and promotion effects by the use of flavonoids, which avoid to operate tedious sample treatment protocols and high-technical ultra-high performance liquid chromatography tandem mass spectrometry (UHPLC-MS/MS) analysis.

### SVM-guided unravelling of reduction/promotion effect of flavonoids on acrylamide formation via the QSAR evaluation

**Figure 5:**
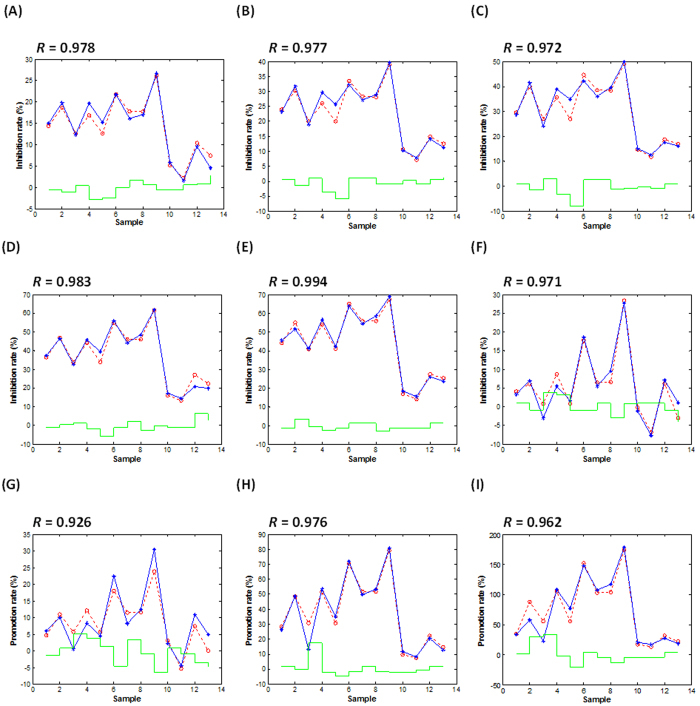
The fitting performance of SVR models as QSAR equations for predicting the reduction or promotion effect on the acrylamide formation when the addition levels of flavonoids were (**A**) 1 μmol/L, (**B**) 5 μmol/L, (**C**) 10 μmol/L, (**D**) 50 μmol/L, (**E**) 100 μmol/L, (**F**) 500 μmol/L, (**G**) 1000 μmol/L, (**H**) 5000 μmol/L, and (**I**) 10000 μmol/L. The red, blue and green lines indicate the predictive data, experimental data and predictive error, respectively. *R*, correlation coefficient for evaluating the fitting performance between experimental and predictive data.

For the QSAR evaluation, the chemical structures of flavonoids exhibiting the lowest energy distribution were initially obtained via the conformational optimization. Then, 1664 molecular descriptors of each compound were calculated for a group of 13 kinds of flavonoids with their effects on acrylamide generation. A genetic algorithm (GA) was next used to choose the best combinations of computed descriptors for associating the structural characteristics of flavonoids with their reduction/promotion effects. As a result, 2 descriptors naming the number of phenol/enol/carboxyl hydroxyl groups (**O-057**) and Moran autocorrelation of lag 7 weighted by mass (**MATS7m**) were selected as variables. Then, the SVR approach was used to establish 9 duplex QSAR models for each selected addition level of flavonoids and predict the reduction or promotion effect of flavonoids (Table 3). Combined with the correlation between experimental and predictive values, currently optimized SVR models as QSAR equations were successful for predicting both reduction and promotion effects on the acrylamide formation by flavonoids in all selected addition levels (*R*: 0.926–0.994, Fig. 5A–I).

The performance of all the final SVR models was checked via applying the 5-fold cross validation technique and investigating the CV-MSE and *Q* (Table 3). As a result, the performance of all SVR models was qualified for both reduction effects by the use of flavonoids (1, 5, 10, 50, 100 and 500 μmol/L, *Q*: 0.831–0.973) and promotion effects by flavonoids (1000, 5000 and 10000 μmol/L, *Q*: 0.798–0.960). To compare with the SVR prediction for current QSAR analyses, both ANN and MLR methods were also successively used for the estimation of inhibition and promotion effects by flavonoids. Using the same molecular descriptors, the fitting performance of both ANN and MLR models when different addition levels of flavonoids were employed (1–10000 μmol/L) were shown in Supplementary Tables 1 and 2, respectively. Considering the comparison results of fitting performance among three models, currently optimized SVR models (*R*: 0.926–0.994) were suitable for predicting both inhibition and promotion effects of flavonoids via QSAR analyses, which were superior to that of both ANN model (*R*: 0.773–0.970) and MLR model (*R*: 0.903–0.973) (Table 3 and Supplementary Tables 1–2).

In view of structural descriptors, **O-057** and **MATS7m** were selected as variables for all established QSAR models, which indicates that both reduction and promotion effects are highly related to characteristic hydroxyls and 2D-level autocorrelation. Considering chemical structures of selected flavonoids, **O-057** represents the number of phenolic hydroxyls in both A and B rings in the structures of flavones, flavonols and isoflavones together with the enolic hydroxyl of C rings (3-hydroxyl) in flavonol structures, but does not refer to the alcoholic hydroxyls in the structures of flavonoid glycosides. Thus, the substitution of 3-O-glycoside reduces **O-057** values. The contribution of **O-057** explains why the reduction/promotion effect by flavonols is better than flavonol 3-O-glycosides even though they possess the same flavone aglycones. **MATS7m** is an indicator of spatial association. Especially, it assumes that the value of a development indicator is not only associated with relevant factors about the lengths of structural fragments, but also related to the values of the indicator observed in adjacent individuals^44^.

QSAR models have been acknowledged as useful tools for the description and prediction of chemical molecular properties^45^. The fundamental principle of QSAR refers to variations in the biological activity of selected chemicals that target an investigated mode of action are associated with changes in their structural, physical and/or chemical properties^46^. In addition to pharmaceutical industry, molecular descriptors^47^, feature selection^48^, modelling^49^ and validation^50^ of QSAR methods have been extensively investigated and applied in the functional prediction of natural products such as flavonoids^51^,^52^. Current study optimized QSAR models for predicting the reduction/promotion effect on the acrylamide formation via the use of different addition levels of flavonoids and found the descriptor appearance of phenolic and enolic hydroxyls in the models during the feature selection. These findings quantitatively demonstrated the key role of phenolic and enolic hydroxyls of flavonoids in their effects on acrylamide formation. Taken together, the use of QSAR models successfully elucidate and predict the effect of flavonoids on the acrylamide formation on a structural evidence basis, which contributes to the structure-activity researches on the chemoprevention of acrylamide.

## Conclusions

In the present work, three kinds of flavonoids with a sequence of addition levels exhibit reduction and promotion effects on acrylamide formation in potato-based asparagine-glucose low-moisture heating model system. The relationship between the addition levels of flavonoids and the dual effects on acrylamide formation seems non-linear dose-dependent. The maximal reduction and promotion effects appear at the addition levels of 100 μmol/L and 10000 μmol/L, which range 15.5–68.8% and 17.1–178.8%, respectively. Using the SVR modelling approach, both reduction and promotion effects by all three kinds of flavonoids at all addition levels on acrylamide formation could be predicted by triple ΔTEAC variables in view of TEAC-dependent properties (*R*: 0.633–0.900) and estimated by optimized QSAR descriptors on a structural basis (*R*: 0.926–0.994). Under the guide of established SVR models, flavonols exhibit stronger dual effects on the acrylamide formation than flavones and isoflavones as well as their O-glycosides derivatives, which may be ascribed to the number and position of phenolic and enolic hydroxyls (**O-057** defined by chemometric parameters) in the chemical skeleton of flavonoids. These observations demonstrated that the SVR modelling approach as a predictive tool in the field of acrylamide-related food safety issues is competent for estimating the effect of natural antioxidants on decreasing the formation of this hazardous toxin. Future work should continuously focus on the optimization and establishment of SVR models for predicting the chemoprevention effect of natural antioxidants on other chemical contaminants during food processing.

## Methods

### Chemicals and materials

Apigenin, luteolin, kaempferol, kaempferol-3-O-glucoside, myricetin, daidzein, genistein, daidzin and genistin (all purity ≥95%) were purchased from Extrasynthese (Lyon, France). Quercetin, quercetin-3-O-glucoside and rutin (all purity ≥95%) were purchased from Sigma-Aldrich (St. Louis, MO, USA). Tricin (purity ≥95%) was chemically synthesized according to our previous publication^53^. Acrylamide, L-asparagine monohydrate, D-(+)-glucose monohydrate, DPPH, ABTS, potassium persulfate and 2,4,6-tri(2-pyridyl)-s-triazine (TPTZ) and 6-hydroxy-2,5,7,8-tetra- methylchromane-2-carboxylic acid (trolox) were purchased from Sigma-Aldrich (St. Louis, MO, USA). Acrylamide-*d*_3_ (isotopic purity ≥99%) was obtained from Cambridge Isotope Laboratories (Andover, MA, USA). Acetonitrile and methanol (HPLC-grade) were purchased from Merck (Kenilworth, NJ, USA) while formic acid (≥96%) was obtained from Tedia (Fairfield, OH, USA). Potato powder (Atlantis variety) was obtained from Sanjiang (Group) Potato Products Co., Ltd. (Lintao, Gansu, China). Water was purified with a Milli-Q system (Millipore, Bedford, USA) and used throughout the sample treatment and instrumental analysis.

### Mimicking the potato-based low-moisture Maillard reaction system

The substrates of current Maillard reaction system were composed of asparagine and glucose with equimolar amounts. This model system was employed to investigate the effect of flavonoids on the acrylamide formation in a low-moisture potato matrix though oven-heating treatments. The mixture of potato powder and the reactant powders of asparagine and glucose was careful grinded to ensure enough surface area for the reaction. Compared to the addition levels of asparagine (1 mmol) and glucose (1 mmol), the original contents of asparagine (0.037 mg) and glucose (0.015 mg) in potato powder (100 mg) were negligible in the present work according to our previous high-performance liquid chromatography (HPLC) analysis^54^.

### Effects of flavonoids on the formation of acrylamide

Addition levels of flavonoids were adopted within a predesigned concentration range in treatment groups, while an equal volume (100  μL) of phosphate buffer (0.1 mol/L, pH 6.80) was employed in the control groups. Working solutions (100 μL) of all selected flavonoids prepared in the above phosphate buffer at a series of concentrations (0.1, 0.5, 1, 5, 10, 50, 100, 500 and 1000 mmol/L) were individually added into the model system in predesigned treatment groups. As a result, the concentrations of flavonoids in final reaction solutions were 1, 5, 10, 50, 100, 500, 1000, 5000 and 10000 μmol/L in different treatment groups. The Maillard reaction was initiated when the oven reached required temperature (180 °C). The fully-mixed reactant powders were then all oven-heated in hermetically sealed hard beakers at 180 °C for 15 min, the optimal heating conditions of when the acrylamide generation reached the maximal level in the control group according to previous work^55^. Finally, the hard- beakers filled with MRP were carefully taken out and then cooled in an ice bath at once to stop any further interaction. The cold MRP were then stored at 4 °C and ready for sample treatment.

### Measurement of acrylamide in MRP by UHPLC-MS/MS

The acrylamide-*d*_3_ internal standard solution (500  μL, 2 μg/mL) was added into the cooled reaction products after their dissolution with the above phosphate buffer (10 mL, 0.1 mol/L, pH 6.8) buffer. The solution was kept oscillating for 10 min in ultrasonic shaker for further homogeneous mixing. The mixture was then extracted with ethyl acetate, followed by a solid-phase extraction clean-up procedure according to previous work^54^. Finally, the collected eluent was sampled for the UHPLC-MS/MS analysis. The chromatographic analyses were conducted on an Acquity ultra-high performance liquid chromatograph equipped with vacuum degasser, sample tray and autosampler (Waters, Milford, MA). The UHPLC separation was achieved with a Waters UPLC BEH C_18_ column (50 mm × 2.1 mm i.d., 1.7 μm) located in an isothermal column compartment. The characteristic precursor and product ions were detected with an electrospray source under the positive mode and quantified by a Quattro Ultima Pt tandem mass spectrometer (Micromass Company Inc., Manchester, UK). The optimal instrumentation parameters for the analysis of acrylamide had been described elsewhere^21^.

### Multiple evaluations of antioxidant properties of MRP

The antioxidant properties of MRP were simultaneously evaluated by three representative antioxidant assays, i.e. DPPH, ABTS and FRAP assays, which had been reported elsewhere^56^,^57^. The results from above antioxidant assays were all expressed in the form of TEAC (μmol trolox per mL sample).

### QSAR evaluation

To start the QSAR evaluation, the chemical structures of flavonoids were drawn via using the Hyperchem software (version 8.0, Hypercube, Inc., Gainesville, FL, USA). The conformational analysis was conducted by molecular mechanics calculations. Considering the presence of O-glycosides in some structures of flavonoids, the Molecular Mechanics (MM+) as variant of both MM2 and assisted model building with energy refinement (AMBER96) force fields were used. Besides, the conformational space of chemical structures was also optimized via using the Austin Model (AM1) semi-empirical method. Detailed parameters of both MM and AM1 semi-empirical methods, including root mean squared gradient value, acceptance energy cutoff and number of conformers, were considered according to previous work^58^. Then, 1664 molecular descriptors of each conformer-stabilized flavonoid were calculated for a group of 13 kinds of flavonoids with their effects on acrylamide generation using the Dragon software (version 5.4, Talete s.r.l., Milano, Italy). The GA was then employed to choose the best combinations of computed descriptors for predicting both inhibition and promotion effects of flavonoids via the GA Toolbox of Matlab software (version R2013a, MathWorks Inc., Natick, MA, USA). The GA started with a population of 50 random models and performed with 150 iterations to evolution with 50% of crossover rate and 1% of mutation rate.

### SVR, ANN and MLR predicting analyses

The SVR, ANN and MLR modelling approaches were comparably used for both antioxidant properties and QSAR evaluation to predict the effect of flavonoids on the acrylamide formation. For the TEAC-dependent evaluation, the training and testing data sets had been initially assigned. The inhibition/promotion rates of acrylamide generation by flavonoids were calculated via the comparison between acrylamide levels in each treatment group and control group. Meanwhile, ΔTEAC_DPPH_, ΔTEAC_ABTS_ and ΔTEAC_FRAP_ values were expressed as three different kinds of TEAC measurements of MRP in each treatment group after deducting respective TEAC values in the control group. Given triplicate measurements for each investigation, there were total 351 groups of experimental data, which were then fit via the SVR approach to predict the inhibition/promotion rates by taking three ΔTEAC values as multiple independent variables. To achieve the uniform distribution of a representative training data subset, a Kennard-Stone algorithm was used for the selection of training and testing data sets via optimizing the Euclidean distance among the vectors of each group of data points^59^. Finally, two-thirds (234 groups of data points) of the results were used for training the predictive models, while the other one-third (117 groups of data points) of the results were used for predicting the effect. All original data were normalized in advance via a minimum-maximum scaling method using the following equation.


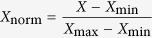


The SVR was performed via the libsvm v2.89 toolbox of Matlab software. The calculation employed ξ-SVR as the SVM type and RBF as the kernel function for the training model. Besides, the other two important parameters, *c* and *g*, were both optimized within a range of 2^−8^–2^8^ via a grid search using the ‘SVMcgForRegress’ function^60^,^61^. A tolerance value of termination criterion (ξ) was set as 0.001 during the search. For current TEAC-dependent prediction work using the SVM architecture, the SVR models were separately established when the addition levels of flavonoids fell into the scopes of 1–100 and 100–10000 μmol/L. The fitting process was achieved via using the ‘svmtrain’ function and the inhibition/promotion rate was then predicted using the ‘svmpredict’ function. The detailed function programs were shown in Supplementary Methods S1-S3. Input data of *X*_1_, *X*_2_ and *X*_3_ represented ΔTEAC_DPPH_, ΔTEAC_ABTS_ and ΔTEAC_FRAP_, respectively, while the output data of *Y* represented reduction/promotion rate of acrylamide formation. The performance of SVR model was evaluated via the fitting outcomes and related statistical variables including RMSE, MAPE and *R*. For the QSAR prediction, the SVR approach was used for establishing 9 duplex QSAR models for each selected addition level of flavonoids via taking 2 molecular descriptors as independent variables, which had been selected by the GA approach. The final SVR models for both TEAC-dependent and QSAR descriptor-dependent predictive studies were internally validated via applying a 5-fold cross validation method and calculating the *Q* and CV-MSE.

To compare the regression efficiency of SVR approach with other acknowledged predictive models, data were alternatively fit via ANN and MLR approaches. For the ANN predicting work, a feed-forward back-propagation training algorithm was used to mimic a network including 1 hidden layer and 10 neurons during the development of machine learning programme. The iteration time was preset to 500, while the learning rate and training target were set to 0.0001 and 0.0002, respectively, which had been optimized according to previous work^29^. The ANN training and predicting process was performed by the nnnetwork toolbox of Matlab software. For the MLR predicting work, a well-known least square iteration algorithm was used to train and establish the MLR equations. Both training and predicting work of MLR were performed by the Statistical Product and Service Solutions (SPSS) software (version 22.0, IBM Corp., Armonk, NY, USA). The performance of both ANN and MLR models was also evaluated via RMSE, MAPE and *R*. The data for describing both inhibition and promotion rates of acrylamide and ΔTEAC amounts in MRP were expressed as mean ± SD (*n* = 3).

## Additional Information

**How to cite this article**: Huang, M. *et al.* Support vector regression-guided unravelling: antioxidant capacity and quantitative structure-activity relationship predict reduction and promotion effects of flavonoids on acrylamide formation. *Sci. Rep.* **6**, 32368; doi: 10.1038/srep32368 (2016).

## Supplementary Material

Supplementary Information

## Figures and Tables

**Table 1:** Association of inhibition and promotion effects of selected flavonoids at different addition levels with their numbers of phenolic and/or 3-enolic hydroxyls.

Addition level of flavonoids (μmol/L)	Linear regression model^a^	*R*^b^	*p*^c^
1	*Y* = 5.287*X* − 3.632	0.963	<0.001***
5	*Y* = 7.015*X* − 0.568	0.943	<0.001***
10	*Y* = 8.609*X* + 1.397	0.927	<0.001***
50	*Y* = 10.92*X* + 0.350	0.946	<0.001***
100	*Y* = 12.54*X* + 1.217	0.939	<0.001***
500	*Y* = 6.391*X* − 15.84	0.919	<0.001***
1000	*Y* = 6.268*X* − 12.22	0.909	<0.001***
5000	*Y* = 17.28*X* − 21.02	0.948	<0.001***
10000	*Y* = 37.53*X* − 55.23	0.902	<0.001***

^a^Linear association of inhibition rate (1, 5, 10, 50, 100 and 500 μmol/L) or promotion rate (1000, 5000 and 10000 μmol/L) of acrylamide (*Y*) with the number of phenolic and/or 3-enolic hydroxyls of flavonoids (*X*) was investigated.

^b^*R*, correlation coefficient.

^c^***p < 0.001.

**Table 2:** Statistical parameters for predicting the inhibition and promotion rates (%) via the use of SVR models compared with the use of ANN and MLR models.

Statistical parameters	Training data				Testing data			
	Experimental	Predicted			Experimental	Predicted		
		SVR	ANN	MLR		SVR	ANN	MLR
Addition level range of flavonoids: 1–100 μmol/L								
	(i) 5-fold cross validation for SVR models (all data)							
CV-MSE	0.072							
*Q*	0.831							
*R*	0.844							
	(ii) Statistical analysis							
Mean	30.2	29.2	28.9	30.2	29.0	29.4	29.6	30.4
SD	16.7	13.6	12.2	13.5	17.3	13.2	12.3	13.2
Median	27.8	28.0	27.5	30.5	27.7	26.9	27.4	29.9
Variance	278.3	184.5	149.7	182.2	297.8	172.8	150.4	173.3
Kurtosis	−0.622	−0.226	−0.033	−0.419	−0.688	−0.717	−0.430	−0.759
Skewness	0.452	0.653	0.668	0.235	0.391	0.561	0.535	0.080
	(iii) Model performance							
RMSE		9.6	9.8	9.8		7.8	8.9	8.0
MAPE		89.2	100.7	73.8		268.6	199.7	194.9
*R*		0.821	0.814	0.809		0.900	0.870	0.898
Addition level range of flavonoids: 100–10000 μmol/L								
	(i) 5-fold cross validation for SVR models (all data)							
CV-MSE	0.094							
*Q*	0.623							
*R*	0.644							
	(ii) Statistical analysis							
Mean	11.5	5.7	5.6	11.5	13.6	4.3	4.0	13.4
SD	49.3	32.1	33.2	31.1	48.5	28.2	30.9	31.0
Median	5.9	7.8	5.8	10.0	7.9	6.2	2.5	9.1
Variance	2425.4	1031.4	1102.7	964.4	2350.6	795.9	952.9	959.9
Kurtosis	1.769	0.582	0.142	−0.783	1.833	−0.752	−0.349	−1.107
Skewness	1.112	0.394	0.506	−0.082	1.044	−0.115	0.277	0.001
	(iii) Model performance							
RMSE		33.2	35.0	38.1		38.5	37.6	36.4
MAPE		145.4	168.9	207.8		157.6	174.1	164.5
*R*		0.753	0.712	0.631		0.633	0.612	0.653

^a^ANN, artificial neural network; CV-MSE, cross validation mean squared error; MAPE, mean absolute percentage error; MLR, multiple linear regression; *Q*, cross validation correlation coefficient; *R*, correlation coefficient; RMSE, root mean square error; SD, standard deviation; SVR, support vector regression.

**Table 3:** SVR parameters and performance for establishing the duplex QSAR models and predicting the inhibition/promotion rates of acrylamide formation based on the selected structural descriptors of flavonoids.

Addition levels (μmol/L)	SVR model variables and parameters^a^						Cross validation^b^	
	Variables	*c*	*g*	nSV	nBSV	*R*	CV-MSE	*Q*
1	**O-057 MATS7m**	8	0.0625	10	6	0.978	0.021	0.968
5		4	0.2500	12	7	0.977	0.025	0.965
10		2	0.5000	12	7	0.972	0.042	0.951
50		2	0.2500	9	6	0.983	0.023	0.973
100		4	0.5000	12	4	0.994	0.027	0.940
500		256	0.1250	11	5	0.971	0.082	0.831
1000		256	0.0039	12	9	0.926	0.094	0.798
5000		8	0.5000	9	2	0.976	0.035	0.960
10000		8	0.5000	11	4	0.962	0.101	0.881

^a^SVR model variables: MATS7m, Moran autocorrelation of lag 7 weighted by mass; O-057, number of phenolic, enolic and carboxyl hydroxyls. Parameters: *c*, cost of ξ-SVR (c); *g*, γ-function of radial basis function (RBF); nSV, number of support vectors; nBSV, number of bounded support vectors.

^b^SVR model performance: CV-MSE, cross validation mean squared error; *Q*, cross validation correlation coefficient; *R*, correlation coefficient of SVR models.
