# Supplementary material for: Support vector regression-guided unravelling: antioxidant capacity and quantitative structure-activity relationship predict reduction and promotion effects of flavonoids on acrylamide formation
Source: Sci Rep. 2016 Sep 2;6:32368. doi: 10.1038/srep32368 (PMC5009353; doi:10.1038/srep32368)
Supplement: Supplementary Information [file srep32368-s1.doc]

**Support vector regression-guided unravelling: antioxidant capacity and quantitative structure-activity relationship predict reduction and promotion effects of flavonoids on acrylamide generation**

Mengmeng Huang^1,2^, Yan Wei^1,2^, Jun Wang^1,2^, Yu Zhang^1,2,*^

^1^ Zhejiang Key Laboratory for Agro-Food Processing, Zhejiang R & D Center for Food Technology and Equipment, Fuli Institute of Food Science, Zhejiang University, Hangzhou 310058, Zhejiang, China.

^2^ Department of Food Science and Nutrition, College of Biosystems Engineering and Food Science, Zhejiang University, Hangzhou 310058, Zhejiang, China.

* Correspondence and requests for materials should be addressed to Y.Z. (email: y_zhang@zju.edu.cn).

**This supplementary file includes Supplementary Figures 1-6, Supplementary Tables 1-2 and Supplementary Methods 1-3.**

**(A)**

**
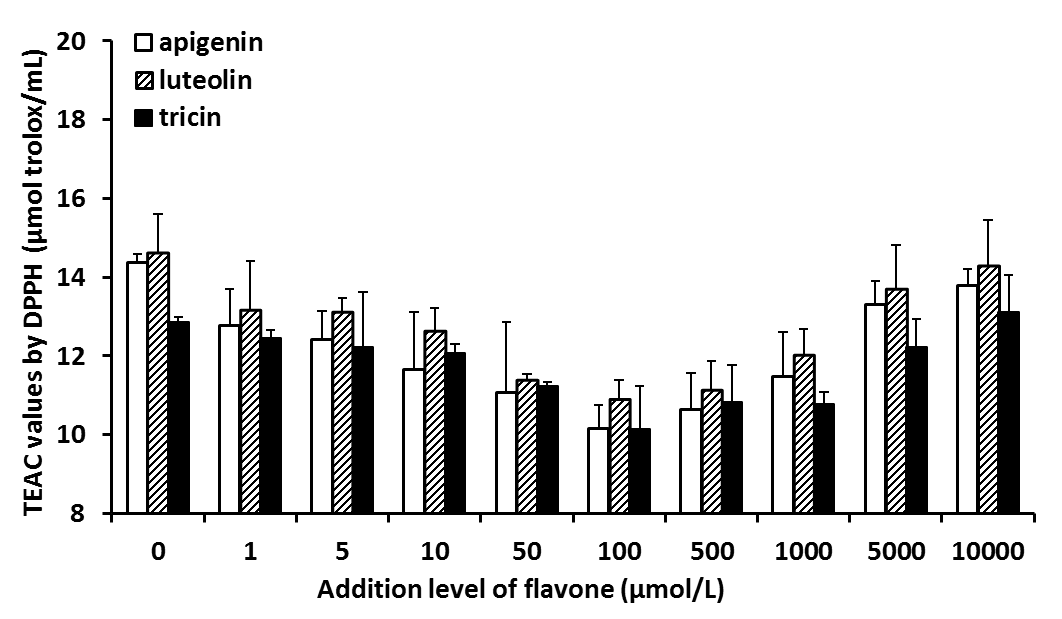
**

**(B)**

**
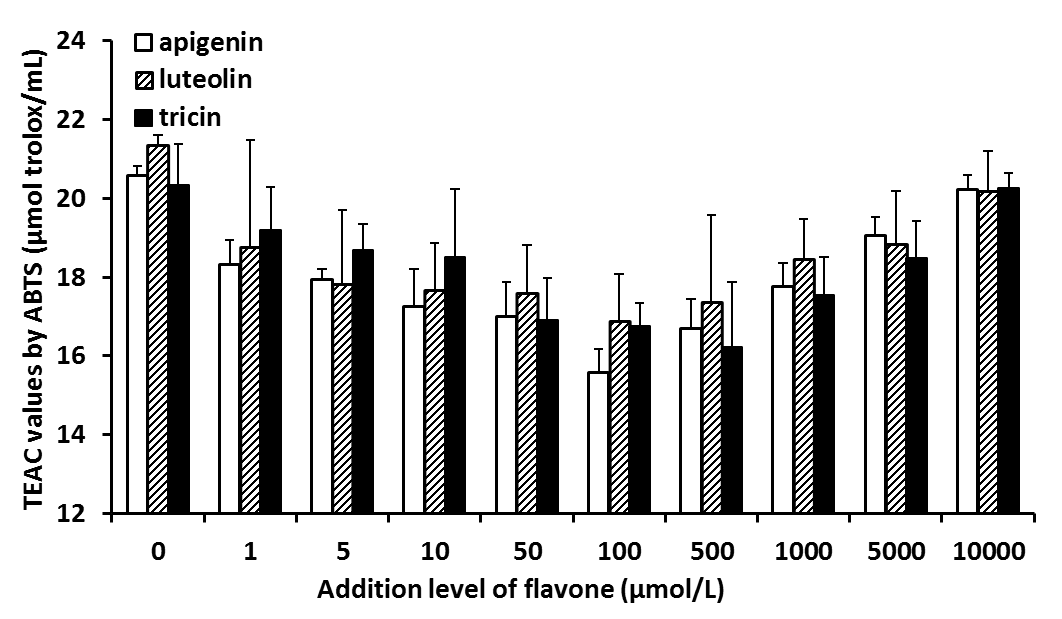
**

**(C)**

**
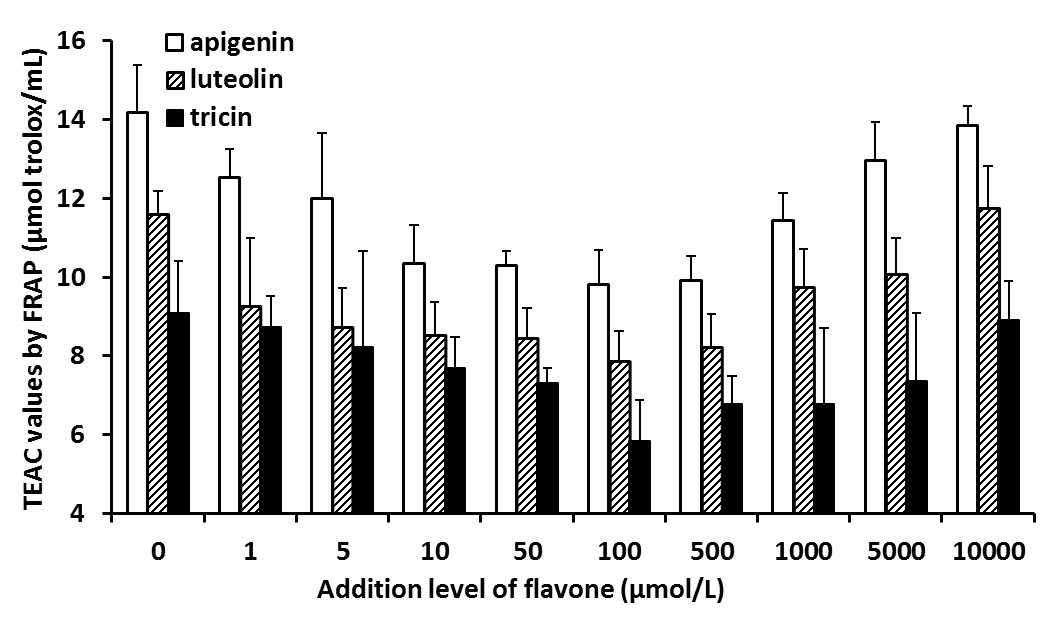
**

**Supplementary Figure 1.** Dose-response correlation between the addition levels of flavones and TEAC values measured by (A) DPPH, (B) ABTS or (C) FRAP assay.

**(A)**

**
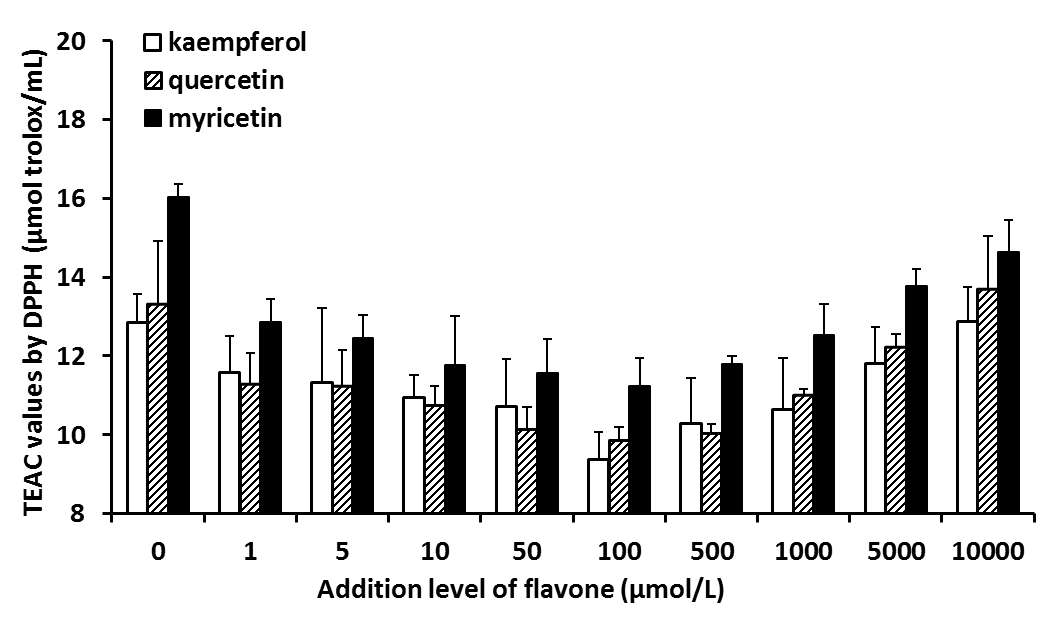
**

**(B)**

**
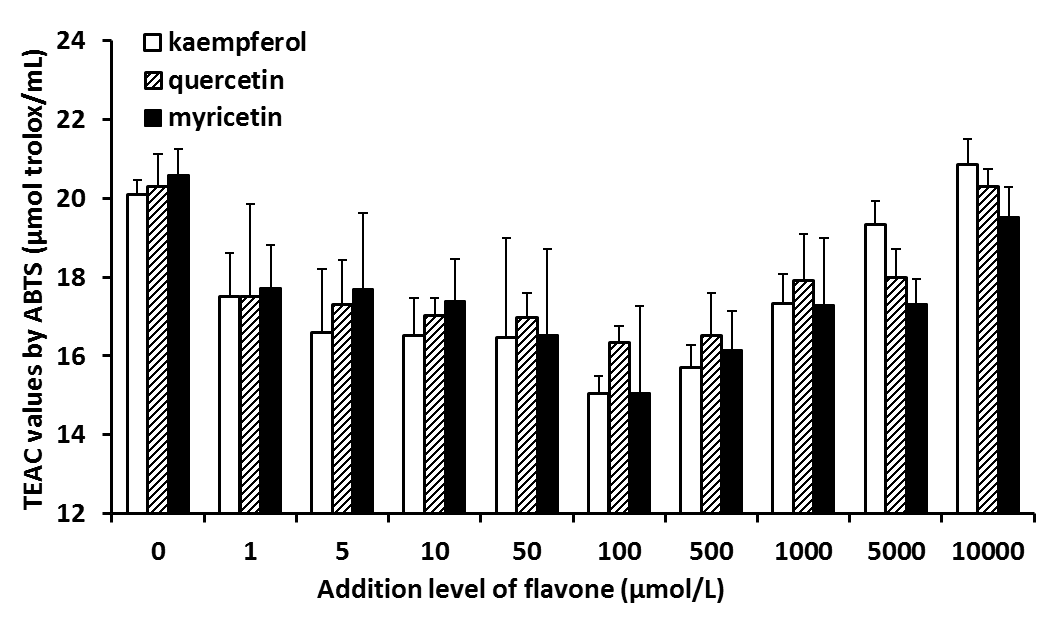
**

**(C)**

**
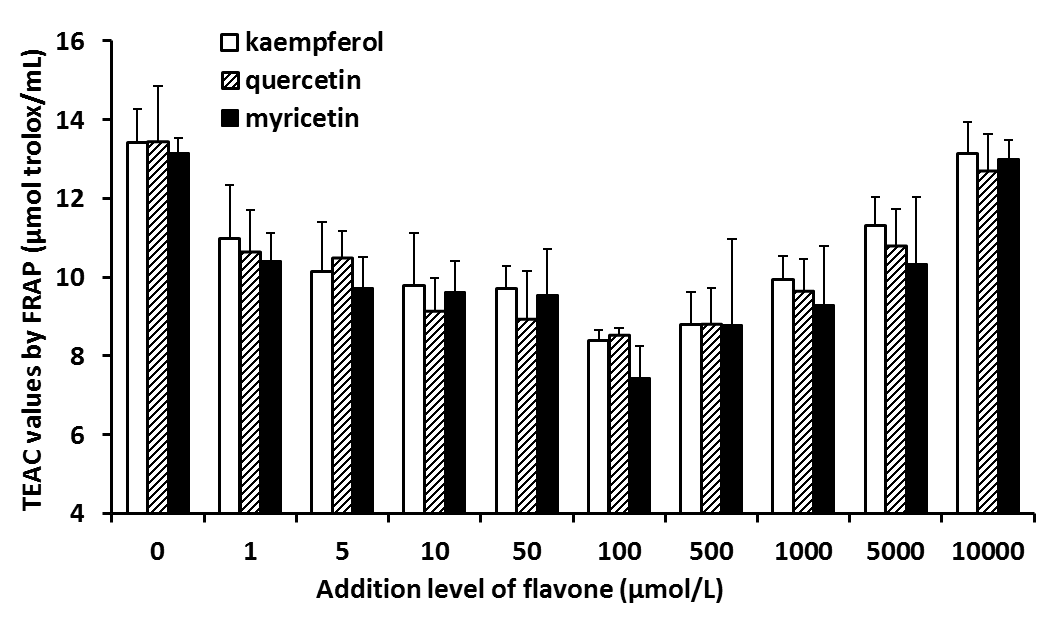
**

**Supplementary Figure 2.** Dose-response correlation between the addition levels of flavonols and TEAC values measured by (A) DPPH, (B) ABTS or (C) FRAP assay.

**(A)**

**
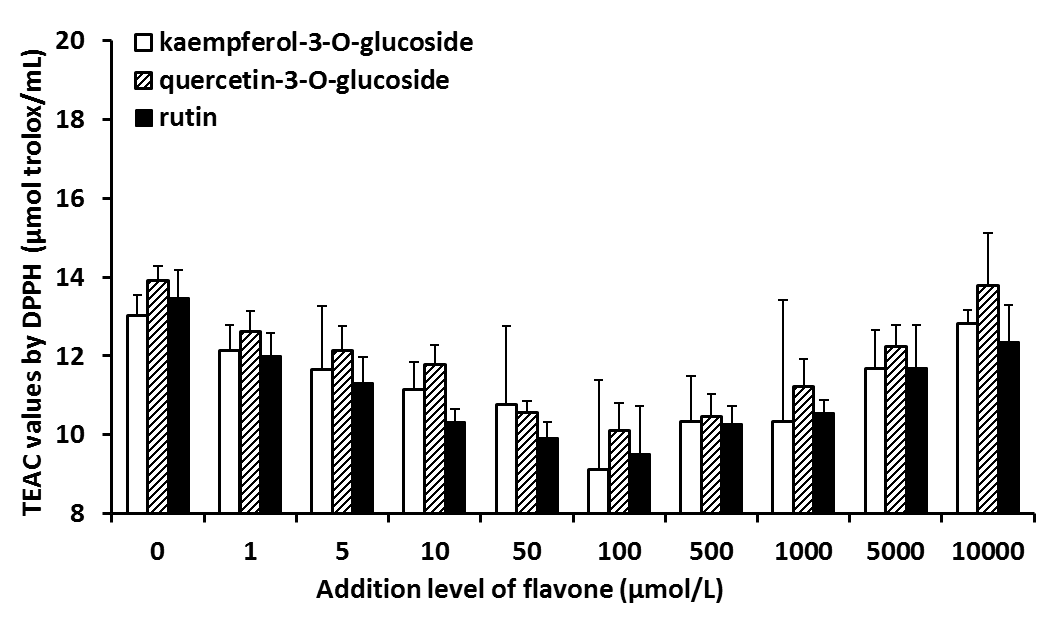
**

**(B)**

**
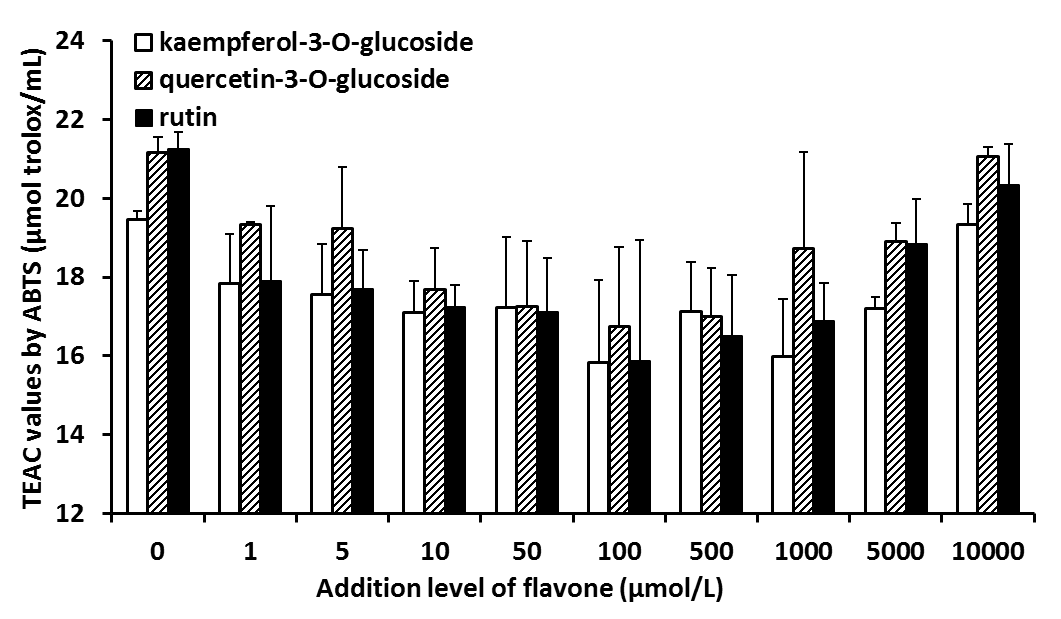
**

**(C)**

**
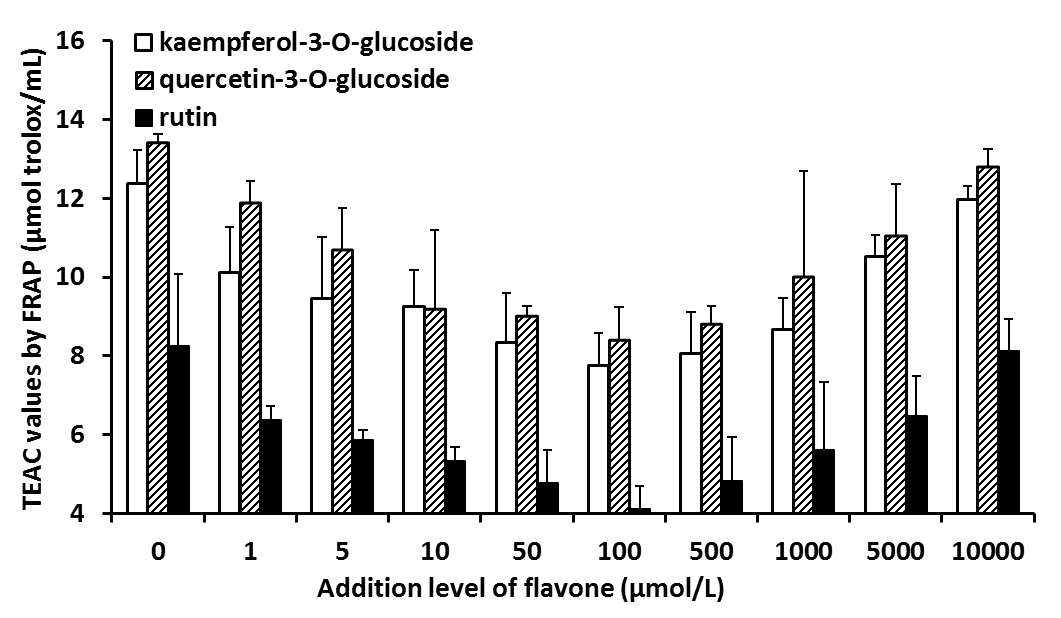
**

**Supplementary Figure 3.** Dose-response correlation between the addition levels of flavonol-3-O-glycosides and TEAC values measured by (A) DPPH, (B) ABTS or (C) FRAP assay.

**(A)**

**
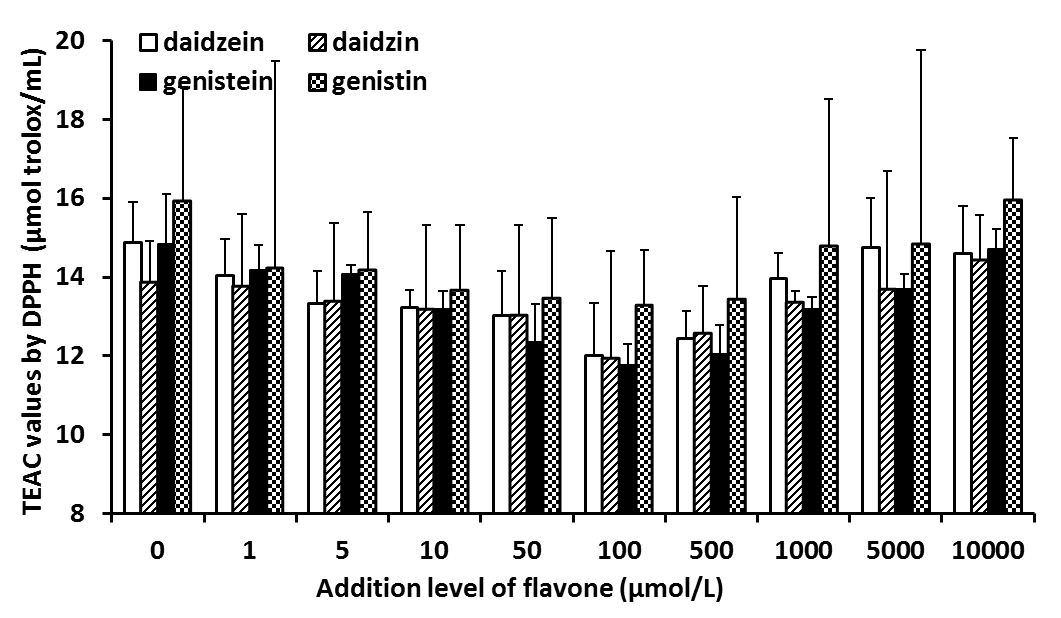
**

**(B)**

**
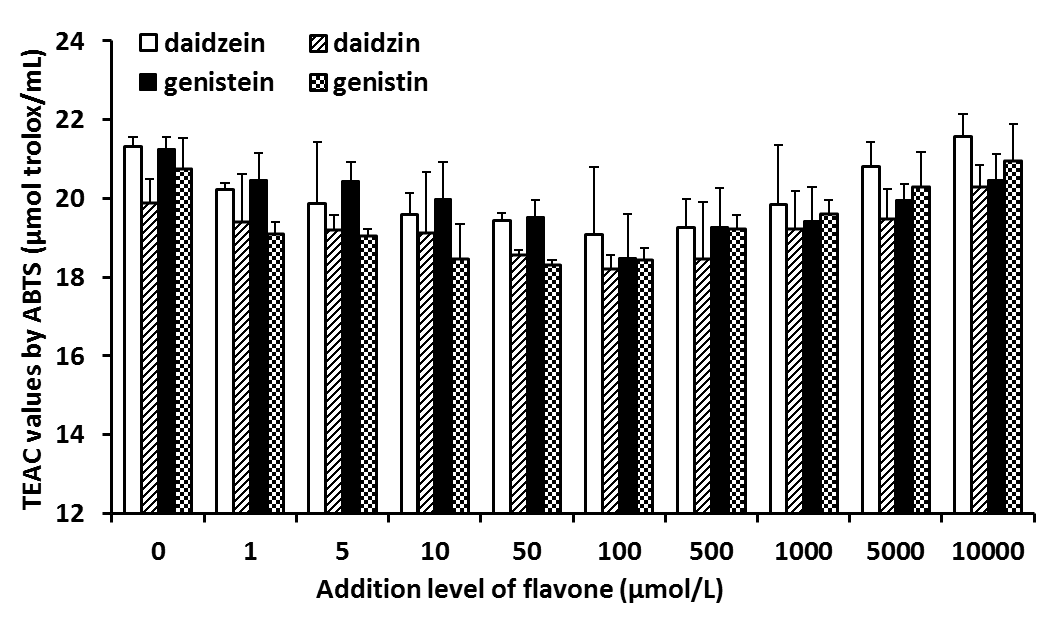
**

**(C)**

**
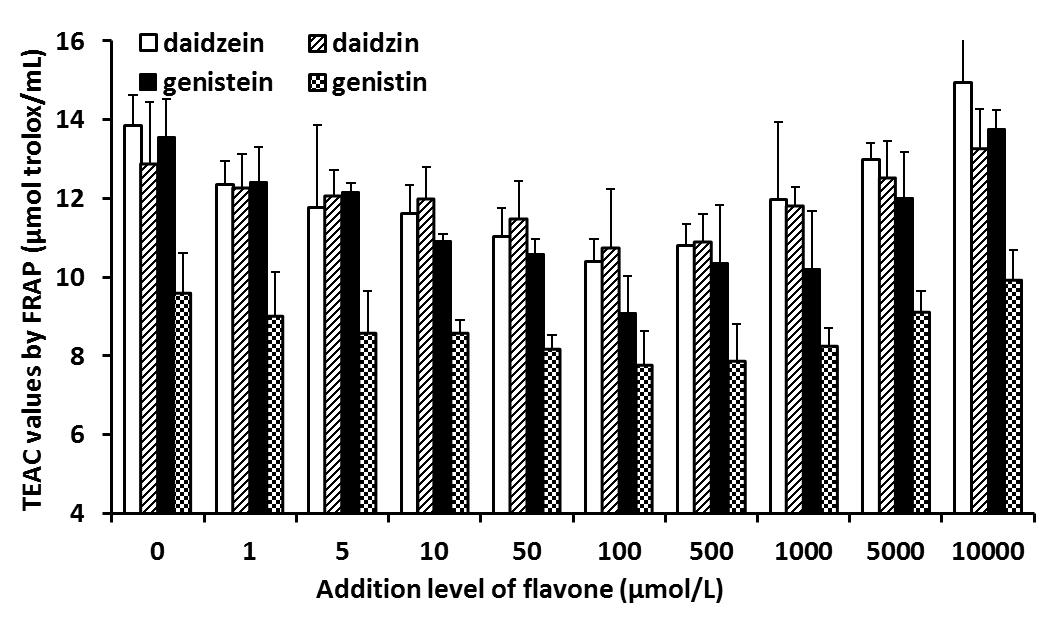
**

**Supplementary Figure 4.** Dose-response correlation between the addition levels of isoflavones and their glycosides and TEAC values measured by (A) DPPH, (B) ABTS or (C) FRAP assay.

**Supplementary Figure 5.** The performance of ANN models for predicting the reduction effect on the acrylamide formation via taking triple antioxidant measurements (DPPH, ABTS and FRAP assays) as variables when the addition levels of flavonoids ranged 1-100 μmol/L. (A-B), The fitting outcomes of (A) training data and (B) testing data via using the nnnetwork toolbox of the Matlab software. The red, blue and green lines indicate the predictive data, experimental data and predictive error, respectively. (C-D), The scatter plots exhibiting the association of predicted data with measured data for (C) training data and (D) testing data using the established ANN model.

**(A)**

**(B)**

**(C)**

**(D)**


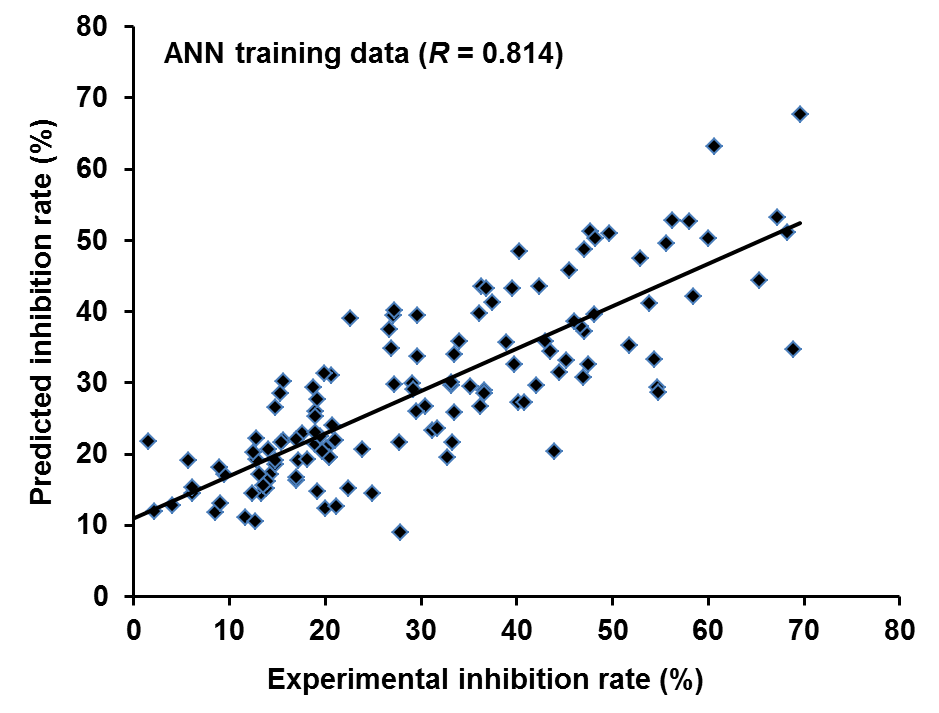

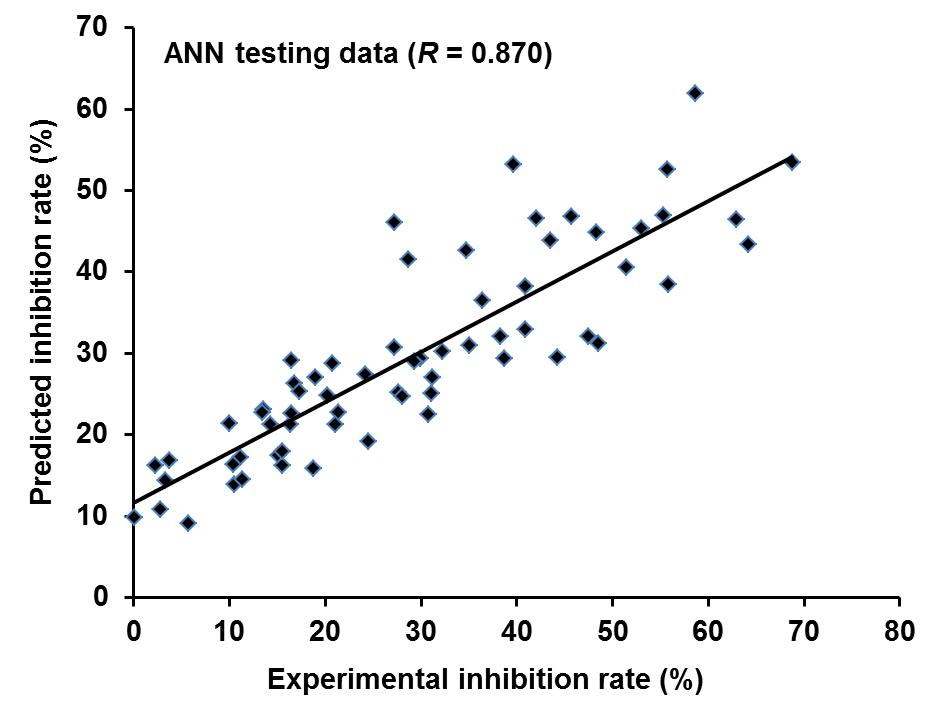


**Supplementary Figure 6.** The performance of ANN models for predicting the promotion effect on the acrylamide formation via taking triple antioxidant measurements (DPPH, ABTS and FRAP assays) as variables when the addition levels of flavonoids ranged 100-10000 μmol/L. (A-B), The fitting outcomes of (A) training data and (B) testing data via using the nnnetwork toolbox of the Matlab software. The red, blue and green lines indicate the predictive data, experimental data and predictive error, respectively. (C-D), The scatter plots exhibiting the association of predicted data with measured data for (C) training data and (D) testing data using the established ANN model.

**(A)**

**(B)**

**(C)**

**(D)**


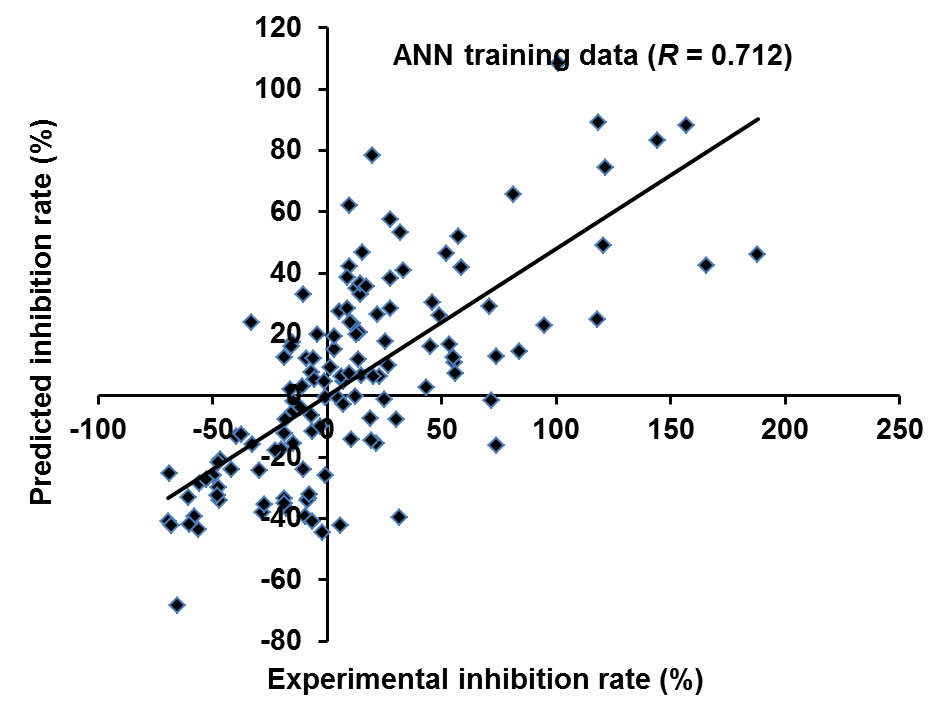

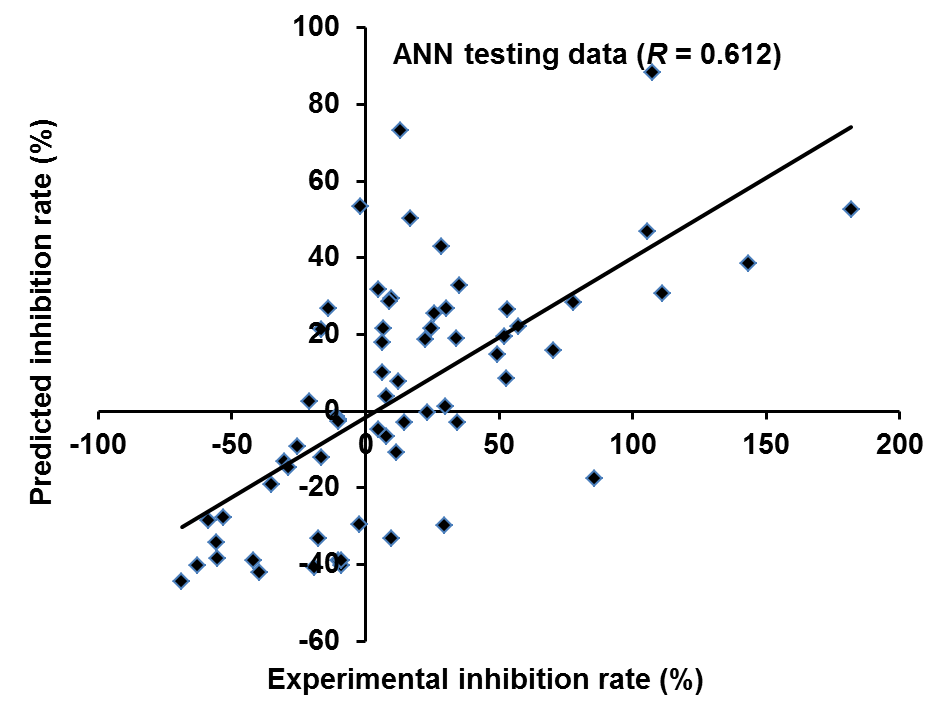


**Supplementary Table 1.** ANN parameters and performance for establishing the multiplex QSAR models and predicting the inhibition/promotion rates of acrylamide formation based on the selected structural descriptors of flavonoids

| Addition levels (μmol/L) | ANN model variables and parameters ^a^ | | | |
| --- | --- | --- | --- | --- |
|  | Variables | *R* | RMSE | MAPE |
| 1 | **O-057**  **MATS7m** | 0.852 | 4.0 | 32.4 |
| 5 |  | 0.810 | 5.8 | 23.5 |
| 10 |  | 0.773 | 8.3 | 25.8 |
| 50 |  | 0.875 | 7.7 | 19.0 |
| 100 |  | 0.908 | 7.9 | 15.0 |
| 500 |  | 0.958 | 2.8 | 66.5 |
| 1000 |  | 0.970 | 2.2 | 26.5 |
| 5000 |  | 0.802 | 17.8 | 59.6 |
| 10000 |  | 0.881 | 25.6 | 38.0 |

^a^ ANN model variables: **MATS7m**, Moran autocorrelation of lag 7 weighted by mass; **O-057**, number of phenolic, enolic and carboxyl hydroxyls.

^b^ SVR model performance: MAPE, mean absolute percentage error; RMSE, root mean squared error; *R*, correlation coefficient.

**Supplementary Table 2. MLR parameters and performance for establishing the multiplex QSAR models and predicting the inhibition/promotion rates of acrylamide formation based on the selected structural descriptors of flavonoids**

| Addition levels (μmol/L) | MLR model variables and parameters^a^ | | | |
| --- | --- | --- | --- | --- |
|  | Variables | *R* | RMSE | MAPE |
| 1 | *Y*_[inhibition]_ = -4.463*X*_[MATS7m]_ + 5.122*X*_[O-057]_ – 2.928 | 0.973 | 1.6 | 15.6 |
| 5 | *Y*_[inhibition]_ = -9.55*X*_[MATS7m]_ + 6.662*X*_[O-057]_ + 0.938 | 0.968 | 2.4 | 11.0 |
| 10 | *Y*_[inhibition]_ = -11.906*X*_[MATS7m]_ + 8.168*X*_[O-057]_ + 3.275 | 0.952 | 3.6 | 12.9 |
| 50 | *Y*_[inhibition]_ = -15.046*X*_[MATS7m]_ + 10.36*X*_[O-057]_ + 2.724 | 0.972 | 3.5 | 10.4 |
| 100 | *Y*_[inhibition]_ = -19.281*X*_[MATS7m]_ + 11.824*X*_[O-057]_ + 4.259 | 0.971 | 4.1 | 8.8 |
| 500 | *Y*_[inhibition]_ = 5.786*X*_[MATS7m]_ + 6.605*X*_[O-057]_ – 16.752 | 0.930 | 3.3 | 87.9 |
| 1000 | *Y*_[promotion]_ = 6.401*X*_[MATS7m]_ + 6.505*X*_[O-057]_ – 13.229 | 0.923 | 3.4 | 98.2 |
| 5000 | *Y*_[promotion]_ = -3.915*X*_[MATS7m]_ + 17.139*X*_[O-057]_ – 20.405 | 0.949 | 7.4 | 30.7 |
| 10000 | *Y*_[promotion]_ = 9.335*X*_[MATS7m]_ + 37.876*X*_[O-057]_ – 56.706 | 0.903 | 22.8 | 54.6 |

^a^ MLR model variables: **MATS7m**, Moran autocorrelation of lag 7 weighted by mass; **O-057**, number of phenolic, enolic and carboxyl hydroxyls.

^b^ MLR model performance: MAPE, mean absolute percentage error; RMSE, root mean squared error; *R*, correlation coefficient.

**Supplementary Method 1.** Source program code of the ‘SVMcgForRegress’ function using the libsvm toolbox of Matlab R2013a.

function [mse,bestc,bestg] = SVMcgForRegress(train_label,train,cmin,cmax,gmin,gmax,v,cstep,gstep,msestep)

%% about the parameters of SVMcg

if nargin < 10

msestep = 0.06;

end

if nargin < 8

cstep = 0.8;

gstep = 0.8;

end

if nargin < 7

v = 5;

end

if nargin < 5

gmax = 8;

gmin = -8;

end

if nargin < 3

cmax = 8;

cmin = -8;

end

%% X:c Y:g cg:acc

[X,Y] = meshgrid(cmin:cstep:cmax,gmin:gstep:gmax);

[m,n] = size(X);

cg = zeros(m,n);

eps = 10^(-4);

%% record acc with different c & g,and find the bestacc with the smallest c

bestc = 0;

bestg = 0;

mse = Inf;

basenum = 2;

for i = 1:m

for j = 1:n

cmd = ['-v ',num2str(v),' -c ',num2str( basenum^X(i,j) ),' -g ',num2str( basenum^Y(i,j) ),' -s 3 -p 0.1'];

cg(i,j) = svmtrain(train_label, train, cmd);

if cg(i,j) < mse

mse = cg(i,j);

bestc = basenum^X(i,j);

bestg = basenum^Y(i,j);

end

if abs( cg(i,j)-mse )<=eps && bestc > basenum^X(i,j)

mse = cg(i,j);

bestc = basenum^X(i,j);

bestg = basenum^Y(i,j);

end

end

end

%% to draw the acc with different c & g

[cg,ps] = mapminmax(cg,0,1);

figure;

[C,h] = contour(X,Y,cg,0:msestep:0.5);

clabel(C,h,'FontSize',10,'Color','r');

xlabel('log2c','FontSize',12);

ylabel('log2g','FontSize',12);

firstline = 'SVR parameter selection (contour view) [GridSearchMethod]';

secondline = ['Best c=',num2str(bestc),' g=',num2str(bestg), ...

' CVmse=',num2str(mse)];

title({firstline;secondline},'Fontsize',12);

grid on;

figure;

meshc(X,Y,cg);

% mesh(X,Y,cg);

% surf(X,Y,cg);

axis([cmin,cmax,gmin,gmax,0,1]);

xlabel('log2c','FontSize',12);

ylabel('log2g','FontSize',12);

zlabel('MSE','FontSize',12);

firstline = 'SVR parameter selection (3D view) [GridSearchMethod]';

secondline = ['Best c=',num2str(bestc),' g=',num2str(bestg), ...

' CVmse=',num2str(mse)];

title({firstline;secondline},'Fontsize',12);

**Supplementary Method 2.** Source program code of the ‘SVMtrain’ function via the C language.

#include <stdio.h>

#include <stdlib.h>

#include <string.h>

#include <ctype.h>

#include "svm.h"

#include "mex.h"

#include "svm_model_matlab.h"

#if MX_API_VER < 0x07030000

typedef int mwIndex;

#endif

#define CMD_LEN 2048

#define Malloc(type,n) (type *)malloc((n)*sizeof(type))

void print_null(const char *s) {}

void exit_with_help()

{

mexPrintf(

"Usage: model = svmtrain(training_label_vector, training_instance_matrix, 'libsvm_options');\n"

"libsvm_options:\n"

"-s svm_type : set type of SVM (default 0)\n"

" 0 -- C-SVC\n"

" 1 -- nu-SVC\n"

" 2 -- one-class SVM\n"

" 3 -- epsilon-SVR\n"

" 4 -- nu-SVR\n"

"-t kernel_type : set type of kernel function (default 2)\n"

" 0 -- linear: u'*v\n"

" 1 -- polynomial: (gamma*u'*v + coef0)^degree\n"

" 2 -- radial basis function: exp(-gamma*|u-v|^2)\n"

" 3 -- sigmoid: tanh(gamma*u'*v + coef0)\n"

" 4 -- precomputed kernel (kernel values in training_instance_matrix)\n"

"-d degree : set degree in kernel function (default 3)\n"

"-g gamma : set gamma in kernel function (default 1/k)\n"

"-r coef0 : set coef0 in kernel function (default 0)\n"

"-c cost : set the parameter C of C-SVC, epsilon-SVR, and nu-SVR (default 1)\n"

"-n nu : set the parameter nu of nu-SVC, one-class SVM, and nu-SVR (default 0.5)\n"

"-p epsilon : set the epsilon in loss function of epsilon-SVR (default 0.1)\n"

"-m cachesize : set cache memory size in MB (default 100)\n"

"-e epsilon : set tolerance of termination criterion (default 0.001)\n"

"-h shrinking : whether to use the shrinking heuristics, 0 or 1 (default 1)\n"

"-b probability_estimates : whether to train a SVC or SVR model for probability estimates, 0 or 1 (default 0)\n"

"-wi weight : set the parameter C of class i to weight*C, for C-SVC (default 1)\n"

"-v n : n-fold cross validation mode\n"

"-q : quiet mode (no outputs)\n"

);

}

// svm arguments

struct svm_parameter param; // set by parse_command_line

struct svm_problem prob; // set by read_problem

struct svm_model *model;

struct svm_node *x_space;

int cross_validation;

int nr_fold;

void (*svm_default_print_string) (const char *) = NULL;

double do_cross_validation()

{

int i;

int total_correct = 0;

double total_error = 0;

double sumv = 0, sumy = 0, sumvv = 0, sumyy = 0, sumvy = 0;

double *target = Malloc(double,prob.l);

double retval = 0.0;

svm_cross_validation(&prob,&param,nr_fold,target);

if(param.svm_type == EPSILON_SVR ||

param.svm_type == NU_SVR)

{

for(i=0;i<prob.l;i++)

{

double y = prob.y[i];

double v = target[i];

total_error += (v-y)*(v-y);

sumv += v;

sumy += y;

sumvv += v*v;

sumyy += y*y;

sumvy += v*y;

}

// mexPrintf("Cross Validation Mean squared error = %g\n",total_error/prob.l);

// mexPrintf("Cross Validation Squared correlation coefficient = %g\n",

// ((prob.l*sumvy-sumv*sumy)*(prob.l*sumvy-sumv*sumy))/

// ((prob.l*sumvv-sumv*sumv)*(prob.l*sumyy-sumy*sumy))

// );

retval = total_error/prob.l;

}

else

{

for(i=0;i<prob.l;i++)

if(target[i] == prob.y[i])

++total_correct;

// mexPrintf("Cross Validation Accuracy = %g%%\n",100.0*total_correct/prob.l);

retval = 100.0*total_correct/prob.l;

}

free(target);

return retval;

}

// nrhs should be 3

int parse_command_line(int nrhs, const mxArray *prhs[], char *model_file_name)

{

int i, argc = 1;

char cmd[CMD_LEN];

char *argv[CMD_LEN/2];

// default values

param.svm_type = C_SVC;

param.kernel_type = RBF;

param.degree = 3;

param.gamma = 0; // 1/k

param.coef0 = 0;

param.nu = 0.5;

param.cache_size = 100;

param.C = 1;

param.eps = 1e-3;

param.p = 0.1;

param.shrinking = 1;

param.probability = 0;

param.nr_weight = 0;

param.weight_label = NULL;

param.weight = NULL;

cross_validation = 0;

// svmtrain loaded only once under matlab

if (svm_default_print_string == NULL)

svm_default_print_string = svm_print_string;

else

svm_print_string = svm_default_print_string;

if(nrhs <= 1)

return 1;

if(nrhs > 2)

{

// put options in argv[]

mxGetString(prhs[2], cmd, mxGetN(prhs[2]) + 1);

if((argv[argc] = strtok(cmd, " ")) != NULL)

while((argv[++argc] = strtok(NULL, " ")) != NULL)

;

}

// parse options

for(i=1;i<argc;i++)

{

if(argv[i][0] != '-') break;

++i;

if(i>=argc && argv[i-1][1] != 'q') // since option -q has no parameter

return 1;

switch(argv[i-1][1])

{

case 's':

param.svm_type = atoi(argv[i]);

break;

case 't':

param.kernel_type = atoi(argv[i]);

break;

case 'd':

param.degree = atoi(argv[i]);

break;

case 'g':

param.gamma = atof(argv[i]);

break;

case 'r':

param.coef0 = atof(argv[i]);

break;

case 'n':

param.nu = atof(argv[i]);

break;

case 'm':

param.cache_size = atof(argv[i]);

break;

case 'c':

param.C = atof(argv[i]);

break;

case 'e':

param.eps = atof(argv[i]);

break;

case 'p':

param.p = atof(argv[i]);

break;

case 'h':

param.shrinking = atoi(argv[i]);

break;

case 'b':

param.probability = atoi(argv[i]);

break;

case 'q':

svm_print_string = &print_null;

i--;

break;

case 'v':

cross_validation = 1;

nr_fold = atoi(argv[i]);

if(nr_fold < 2)

{

mexPrintf("n-fold cross validation: n must >= 2\n");

return 1;

}

break;

case 'w':

++param.nr_weight;

param.weight_label = (int *)realloc(param.weight_label,sizeof(int)*param.nr_weight);

param.weight = (double *)realloc(param.weight,sizeof(double)*param.nr_weight);

param.weight_label[param.nr_weight-1] = atoi(&argv[i-1][2]);

param.weight[param.nr_weight-1] = atof(argv[i]);

break;

default:

mexPrintf("Unknown option -%c\n", argv[i-1][1]);

return 1;

}

}

return 0;

}

// read in a problem (in svmlight format)

int read_problem_dense(const mxArray *label_vec, const mxArray *instance_mat)

{

int i, j, k;

int elements, max_index, sc, label_vector_row_num;

double *samples, *labels;

prob.x = NULL;

prob.y = NULL;

x_space = NULL;

labels = mxGetPr(label_vec);

samples = mxGetPr(instance_mat);

sc = (int)mxGetN(instance_mat);

elements = 0;

// the number of instance

prob.l = (int)mxGetM(instance_mat);

label_vector_row_num = (int)mxGetM(label_vec);

if(label_vector_row_num!=prob.l)

{

mexPrintf("Length of label vector does not match # of instances.\n");

return -1;

}

if(param.kernel_type == PRECOMPUTED)

elements = prob.l * (sc + 1);

else

{

for(i = 0; i < prob.l; i++)

{

for(k = 0; k < sc; k++)

if(samples[k * prob.l + i] != 0)

elements++;

// count the '-1' element

elements++;

}

}

prob.y = Malloc(double,prob.l);

prob.x = Malloc(struct svm_node *,prob.l);

x_space = Malloc(struct svm_node, elements);

max_index = sc;

j = 0;

for(i = 0; i < prob.l; i++)

{

prob.x[i] = &x_space[j];

prob.y[i] = labels[i];

for(k = 0; k < sc; k++)

{

if(param.kernel_type == PRECOMPUTED || samples[k * prob.l + i] != 0)

{

x_space[j].index = k + 1;

x_space[j].value = samples[k * prob.l + i];

j++;

}

}

x_space[j++].index = -1;

}

if(param.gamma == 0 && max_index > 0)

param.gamma = 1.0/max_index;

if(param.kernel_type == PRECOMPUTED)

for(i=0;i<prob.l;i++)

{

if((int)prob.x[i][0].value <= 0 || (int)prob.x[i][0].value > max_index)

{

mexPrintf("Wrong input format: sample_serial_number out of range\n");

return -1;

}

}

return 0;

}

int read_problem_sparse(const mxArray *label_vec, const mxArray *instance_mat)

{

int i, j, k, low, high;

mwIndex *ir, *jc;

int elements, max_index, num_samples, label_vector_row_num;

double *samples, *labels;

mxArray *instance_mat_col; // transposed instance sparse matrix

prob.x = NULL;

prob.y = NULL;

x_space = NULL;

// transpose instance matrix

{

mxArray *prhs[1], *plhs[1];

prhs[0] = mxDuplicateArray(instance_mat);

if(mexCallMATLAB(1, plhs, 1, prhs, "transpose"))

{

mexPrintf("Error: cannot transpose training instance matrix\n");

return -1;

}

instance_mat_col = plhs[0];

mxDestroyArray(prhs[0]);

}

// each column is one instance

labels = mxGetPr(label_vec);

samples = mxGetPr(instance_mat_col);

ir = mxGetIr(instance_mat_col);

jc = mxGetJc(instance_mat_col);

num_samples = (int)mxGetNzmax(instance_mat_col);

// the number of instance

prob.l = (int)mxGetN(instance_mat_col);

label_vector_row_num = (int)mxGetM(label_vec);

if(label_vector_row_num!=prob.l)

{

mexPrintf("Length of label vector does not match # of instances.\n");

return -1;

}

elements = num_samples + prob.l;

max_index = (int)mxGetM(instance_mat_col);

prob.y = Malloc(double,prob.l);

prob.x = Malloc(struct svm_node *,prob.l);

x_space = Malloc(struct svm_node, elements);

j = 0;

for(i=0;i<prob.l;i++)

{

prob.x[i] = &x_space[j];

prob.y[i] = labels[i];

low = (int)jc[i], high = (int)jc[i+1];

for(k=low;k<high;k++)

{

x_space[j].index = (int)ir[k] + 1;

x_space[j].value = samples[k];

j++;

}

x_space[j++].index = -1;

}

if(param.gamma == 0 && max_index > 0)

param.gamma = 1.0/max_index;

return 0;

}

static void fake_answer(mxArray *plhs[])

{

plhs[0] = mxCreateDoubleMatrix(0, 0, mxREAL);

}

// Interface function of matlab

// now assume prhs[0]: label prhs[1]: features

void mexFunction( int nlhs, mxArray *plhs[],

int nrhs, const mxArray *prhs[] )

{

const char *error_msg;

// fix random seed to have same results for each run

// (for cross validation and probability estimation)

srand(1);

// Transform the input Matrix to libsvm format

if(nrhs > 0 && nrhs < 4)

{

int err;

if(!mxIsDouble(prhs[0]) || !mxIsDouble(prhs[1])) {

mexPrintf("Error: label vector and instance matrix must be double\n");

fake_answer(plhs);

return;

}

if(parse_command_line(nrhs, prhs, NULL))

{

exit_with_help();

svm_destroy_param(&param);

fake_answer(plhs);

return;

}

if(mxIsSparse(prhs[1]))

{

if(param.kernel_type == PRECOMPUTED)

{

// precomputed kernel requires dense matrix, so we make one

mxArray *rhs[1], *lhs[1];

rhs[0] = mxDuplicateArray(prhs[1]);

if(mexCallMATLAB(1, lhs, 1, rhs, "full"))

{

mexPrintf("Error: cannot generate a full training instance matrix\n");

svm_destroy_param(&param);

fake_answer(plhs);

return;

}

err = read_problem_dense(prhs[0], lhs[0]);

mxDestroyArray(lhs[0]);

mxDestroyArray(rhs[0]);

}

else

err = read_problem_sparse(prhs[0], prhs[1]);

}

else

err = read_problem_dense(prhs[0], prhs[1]);

// svmtrain's original code

error_msg = svm_check_parameter(&prob, &param);

if(err || error_msg)

{

if (error_msg != NULL)

mexPrintf("Error: %s\n", error_msg);

svm_destroy_param(&param);

free(prob.y);

free(prob.x);

free(x_space);

fake_answer(plhs);

return;

}

if(cross_validation)

{

double *ptr;

plhs[0] = mxCreateDoubleMatrix(1, 1, mxREAL);

ptr = mxGetPr(plhs[0]);

ptr[0] = do_cross_validation();

}

else

{

int nr_feat = (int)mxGetN(prhs[1]);

const char *error_msg;

model = svm_train(&prob, &param);

error_msg = model_to_matlab_structure(plhs, nr_feat, model);

if(error_msg)

mexPrintf("Error: can't convert libsvm model to matrix structure: %s\n", error_msg);

svm_destroy_model(model);

}

svm_destroy_param(&param);

free(prob.y);

free(prob.x);

free(x_space);

}

else

{

exit_with_help();

fake_answer(plhs);

return;

}

}

**Supplementary Method 3.** Source program code of the ‘SVMpredict’ function via the C language.

#include <stdio.h>

#include <stdlib.h>

#include <string.h>

#include "svm.h"

#include "mex.h"

#include "svm_model_matlab.h"

#if MX_API_VER < 0x07030000

typedef int mwIndex;

#endif

#define CMD_LEN 2048

void read_sparse_instance(const mxArray *prhs, int index, struct svm_node *x)

{

int i, j, low, high;

mwIndex *ir, *jc;

double *samples;

ir = mxGetIr(prhs);

jc = mxGetJc(prhs);

samples = mxGetPr(prhs);

// each column is one instance

j = 0;

low = (int)jc[index], high = (int)jc[index+1];

for(i=low;i<high;i++)

{

x[j].index = (int)ir[i] + 1;

x[j].value = samples[i];

j++;

}

x[j].index = -1;

}

static void fake_answer(mxArray *plhs[])

{

plhs[0] = mxCreateDoubleMatrix(0, 0, mxREAL);

plhs[1] = mxCreateDoubleMatrix(0, 0, mxREAL);

plhs[2] = mxCreateDoubleMatrix(0, 0, mxREAL);

}

void predict(mxArray *plhs[], const mxArray *prhs[], struct svm_model *model, const int predict_probability)

{

int label_vector_row_num, label_vector_col_num;

int feature_number, testing_instance_number;

int instance_index;

double *ptr_instance, *ptr_label, *ptr_predict_label;

double *ptr_prob_estimates, *ptr_dec_values, *ptr;

struct svm_node *x;

mxArray *pplhs[1]; // transposed instance sparse matrix

int correct = 0;

int total = 0;

double error = 0;

double sump = 0, sumt = 0, sumpp = 0, sumtt = 0, sumpt = 0;

int svm_type=svm_get_svm_type(model);

int nr_class=svm_get_nr_class(model);

double *prob_estimates=NULL;

// prhs[1] = testing instance matrix

feature_number = (int)mxGetN(prhs[1]);

testing_instance_number = (int)mxGetM(prhs[1]);

label_vector_row_num = (int)mxGetM(prhs[0]);

label_vector_col_num = (int)mxGetN(prhs[0]);

if(label_vector_row_num!=testing_instance_number)

{

mexPrintf("Length of label vector does not match # of instances.\n");

fake_answer(plhs);

return;

}

if(label_vector_col_num!=1)

{

mexPrintf("label (1st argument) should be a vector (# of column is 1).\n");

fake_answer(plhs);

return;

}

ptr_instance = mxGetPr(prhs[1]);

ptr_label = mxGetPr(prhs[0]);

// transpose instance matrix

if(mxIsSparse(prhs[1]))

{

if(model->param.kernel_type == PRECOMPUTED)

{

// precomputed kernel requires dense matrix, so we make one

mxArray *rhs[1], *lhs[1];

rhs[0] = mxDuplicateArray(prhs[1]);

if(mexCallMATLAB(1, lhs, 1, rhs, "full"))

{

mexPrintf("Error: cannot full testing instance matrix\n");

fake_answer(plhs);

return;

}

ptr_instance = mxGetPr(lhs[0]);

mxDestroyArray(rhs[0]);

}

else

{

mxArray *pprhs[1];

pprhs[0] = mxDuplicateArray(prhs[1]);

if(mexCallMATLAB(1, pplhs, 1, pprhs, "transpose"))

{

mexPrintf("Error: cannot transpose testing instance matrix\n");

fake_answer(plhs);

return;

}

}

}

if(predict_probability)

{

if(svm_type==NU_SVR || svm_type==EPSILON_SVR)

mexPrintf("Prob. model for test data: target value = predicted value + z,\nz: Laplace distribution e^(-|z|/sigma)/(2sigma),sigma=%g\n",svm_get_svr_probability(model));

else

prob_estimates = (double *) malloc(nr_class*sizeof(double));

}

plhs[0] = mxCreateDoubleMatrix(testing_instance_number, 1, mxREAL);

if(predict_probability)

{

// prob estimates are in plhs[2]

if(svm_type==C_SVC || svm_type==NU_SVC)

plhs[2] = mxCreateDoubleMatrix(testing_instance_number, nr_class, mxREAL);

else

plhs[2] = mxCreateDoubleMatrix(0, 0, mxREAL);

}

else

{

// decision values are in plhs[2]

if(svm_type == ONE_CLASS ||

svm_type == EPSILON_SVR ||

svm_type == NU_SVR)

plhs[2] = mxCreateDoubleMatrix(testing_instance_number, 1, mxREAL);

else

plhs[2] = mxCreateDoubleMatrix(testing_instance_number, nr_class*(nr_class-1)/2, mxREAL);

}

ptr_predict_label = mxGetPr(plhs[0]);

ptr_prob_estimates = mxGetPr(plhs[2]);

ptr_dec_values = mxGetPr(plhs[2]);

x = (struct svm_node*)malloc((feature_number+1)*sizeof(struct svm_node) );

for(instance_index=0;instance_index<testing_instance_number;instance_index++)

{

int i;

double target_label, predict_label;

target_label = ptr_label[instance_index];

if(mxIsSparse(prhs[1]) && model->param.kernel_type != PRECOMPUTED) // prhs[1]^T is still sparse

read_sparse_instance(pplhs[0], instance_index, x);

else

{

for(i=0;i<feature_number;i++)

{

x[i].index = i+1;

x[i].value = ptr_instance[testing_instance_number*i+instance_index];

}

x[feature_number].index = -1;

}

if(predict_probability)

{

if(svm_type==C_SVC || svm_type==NU_SVC)

{

predict_label = svm_predict_probability(model, x, prob_estimates);

ptr_predict_label[instance_index] = predict_label;

for(i=0;i<nr_class;i++)

ptr_prob_estimates[instance_index + i * testing_instance_number] = prob_estimates[i];

} else {

predict_label = svm_predict(model,x);

ptr_predict_label[instance_index] = predict_label;

}

}

else

{

predict_label = svm_predict(model,x);

ptr_predict_label[instance_index] = predict_label;

if(svm_type == ONE_CLASS ||

svm_type == EPSILON_SVR ||

svm_type == NU_SVR)

{

double res;

svm_predict_values(model, x, &res);

ptr_dec_values[instance_index] = res;

}

else

{

double *dec_values = (double *) malloc(sizeof(double) * nr_class*(nr_class-1)/2);

svm_predict_values(model, x, dec_values);

for(i=0;i<(nr_class*(nr_class-1))/2;i++)

ptr_dec_values[instance_index + i * testing_instance_number] = dec_values[i];

free(dec_values);

}

}

if(predict_label == target_label)

++correct;

error += (predict_label-target_label)*(predict_label-target_label);

sump += predict_label;

sumt += target_label;

sumpp += predict_label*predict_label;

sumtt += target_label*target_label;

sumpt += predict_label*target_label;

++total;

}

if(svm_type==NU_SVR || svm_type==EPSILON_SVR)

{

mexPrintf("Mean squared error = %g (regression)\n",error/total);

mexPrintf("Squared correlation coefficient = %g (regression)\n",

((total*sumpt-sump*sumt)*(total*sumpt-sump*sumt))/

((total*sumpp-sump*sump)*(total*sumtt-sumt*sumt))

);

}

else

mexPrintf("Accuracy = %g%% (%d/%d) (classification)\n",

(double)correct/total*100,correct,total);

// return accuracy, mean squared error, squared correlation coefficient

plhs[1] = mxCreateDoubleMatrix(3, 1, mxREAL);

ptr = mxGetPr(plhs[1]);

ptr[0] = (double)correct/total*100;

ptr[1] = error/total;

ptr[2] = ((total*sumpt-sump*sumt)*(total*sumpt-sump*sumt))/

((total*sumpp-sump*sump)*(total*sumtt-sumt*sumt));

free(x);

if(prob_estimates != NULL)

free(prob_estimates);

}

void exit_with_help()

{

mexPrintf(

"Usage: [predicted_label, accuracy, decision_values/prob_estimates] = svmpredict(testing_label_vector, testing_instance_matrix, model, 'libsvm_options')\n"

"Parameters:\n"

" model: SVM model structure from svmtrain.\n"

" libsvm_options:\n"

" -b probability_estimates: whether to predict probability estimates, 0 or 1 (default 0); one-class SVM not supported yet\n"

"Returns:\n"

" predicted_label: SVM prediction output vector.\n"

" accuracy: a vector with accuracy, mean squared error, squared correlation coefficient.\n"

" prob_estimates: If selected, probability estimate vector.\n"

);

}

void mexFunction( int nlhs, mxArray *plhs[],

int nrhs, const mxArray *prhs[] )

{

int prob_estimate_flag = 0;

struct svm_model *model;

if(nrhs > 4 || nrhs < 3)

{

exit_with_help();

fake_answer(plhs);

return;

}

if(!mxIsDouble(prhs[0]) || !mxIsDouble(prhs[1])) {

mexPrintf("Error: label vector and instance matrix must be double\n");

fake_answer(plhs);

return;

}

if(mxIsStruct(prhs[2]))

{

const char *error_msg;

// parse options

if(nrhs==4)

{

int i, argc = 1;

char cmd[CMD_LEN], *argv[CMD_LEN/2];

// put options in argv[]

mxGetString(prhs[3], cmd, mxGetN(prhs[3]) + 1);

if((argv[argc] = strtok(cmd, " ")) != NULL)

while((argv[++argc] = strtok(NULL, " ")) != NULL)

;

for(i=1;i<argc;i++)

{

if(argv[i][0] != '-') break;

if(++i>=argc)

{

exit_with_help();

fake_answer(plhs);

return;

}

switch(argv[i-1][1])

{

case 'b':

prob_estimate_flag = atoi(argv[i]);

break;

default:

mexPrintf("Unknown option: -%c\n", argv[i-1][1]);

exit_with_help();

fake_answer(plhs);

return;

}

}

}

model = matlab_matrix_to_model(prhs[2], &error_msg);

if (model == NULL)

{

mexPrintf("Error: can't read model: %s\n", error_msg);

fake_answer(plhs);

return;

}

if(prob_estimate_flag)

{

if(svm_check_probability_model(model)==0)

{

mexPrintf("Model does not support probabiliy estimates\n");

fake_answer(plhs);

svm_destroy_model(model);

return;

}

}

else

{

if(svm_check_probability_model(model)!=0)

printf("Model supports probability estimates, but disabled in predicton.\n");

}

predict(plhs, prhs, model, prob_estimate_flag);

// destroy model

svm_destroy_model(model);

}

else

{

mexPrintf("model file should be a struct array\n");

fake_answer(plhs);

}

return;

}
